# Integrin Trafficking, Fibronectin Architecture, and Glomerular Injury upon Adiponectin Receptor 1 Depletion

**DOI:** 10.1681/ASN.0000000611

**Published:** 2025-01-28

**Authors:** Sonja Lindfors, Constanze Schmotz, Dominik Lewandowski, Annika Hau, Leena Saikko, Eero Lehtonen, Ville Majaniemi, Minna Karhe, Jette-Britt Naams, Harry Nisen, Jukka Tienari, Moin A. Saleem, Katharina Pfeil, Heiko Bugger, Kirsi H. Pietiläinen, Tuomas Mirtti, Krzysztof Palczewski, Sanna Lehtonen

**Affiliations:** 1 Research Program for Clinical and Molecular Metabolism, Faculty of Medicine, University of Helsinki, Helsinki, Finland; 2 Gavin Herbert Eye Institute-Center for Translational Vision Research, Department of Ophthalmology, University of California, Irvine, Irvine, California; 3 Department of Pathology, University of Helsinki, Helsinki, Finland; 4 Abdominal Center, Urology, University of Helsinki and Helsinki University Hospital, Helsinki, Finland; 5 Department of Pathology, University of Helsinki and Helsinki University Hospital, Helsinki, Finland; 6 Bristol Renal, Translational Health Sciences, Bristol Medical School, University of Bristol, Bristol, United Kingdom; 7 Department of Cardiology, Medical University of Graz, Graz, Austria; 8 Obesity Research Unit, Faculty of Medicine, University of Helsinki, Helsinki, Finland; 9 Healthy Weight Hub, Abdominal Center, Helsinki University Hospital and University of Helsinki, Helsinki, Finland; 10 Research Program in Systems Oncology, Faculty of Medicine, University of Helsinki, Helsinki, Finland; 11 Department of Physiology and Biophysics, University of California, Irvine, Irvine, California; 12 Department of Chemistry, University of California, Irvine, Irvine, California; 13 Department of Molecular Biology and Biochemistry, University of California, Irvine, Irvine, California

**Keywords:** glomerulus, podocyte, diabetic kidney disease

## Abstract

**Key Points:**

- Glomerular expression of adiponectin receptor 1 (AdipoR1) was lower in people with type 2 diabetes and correlates with podocyte loss.
- AdipoR1 knockout induced glomerular injury and fibrosis in mice, predominantly in males.
- AdipoR1 knockdown podocytes showed impaired trafficking of active integrin *β*1, fibronectin accumulation, impaired adhesion, and increased apoptosis.

**Background:**

Deficiency of adiponectin and its downstream signaling may contribute to the pathogenesis of kidney injury in type 2 diabetes. Adiponectin activates intracellular signaling using adiponectin receptor 1 (AdipoR1) and adiponectin receptor 2, but the role of adiponectin receptor–mediated signaling in glomerular injury in type 2 diabetes remains unknown.

**Methods:**

The expression of AdipoR1 in the kidneys of people with type 2 diabetes and the expression of podocyte proteins or injury markers in the kidneys of AdipoR1 knockout (AdipoR1-KO) mice and immortalized AdipoR1-deficient human podocytes were investigated by immunohistochemistry and immunoblotting. The functional role of AdipoR1 was studied in AdipoR1-deficient podocytes by performing assays for apoptosis, cytokine secretion, mechanical stress, adhesion, and endocytic trafficking.

**Results:**

Glomerular AdipoR1 expression was lower in type 2 diabetes and associated kidney disease, correlating with higher body mass index and podocyte loss. Male AdipoR1-KO mice showed typical signs of early diabetic kidney disease, including albuminuria, glomerular structural abnormalities, and lower expression of central podocyte proteins; females were less affected. Podocyte apoptosis increased in female and male AdipoR1-KO mice, and excessive podocyte loss, potentially due to detachment, was detected in males. AdipoR1 deficiency impaired the yes-associated protein–mediated mechanoresponse and induced accumulation of the extracellular matrix (ECM) protein fibronectin in the glomeruli *in vivo* and podocytes *in vitro*. Functionally, AdipoR1 deficiency impaired endocytosis of the ECM receptor active integrin *β*1, disturbed focal adhesion turnover, and remodulated podocyte-derived ECM, thereby reducing podocyte adhesion.

**Conclusions:**

AdipoR1 deficiency in mice resulted in the development of kidney injury predominantly in males. Mechanistically, AdipoR1 loss in podocytes impaired endocytosis of active integrin *β*1, which plausibly compromised focal adhesion dynamics, disturbed fibronectin matrix turnover, and hindered podocyte adhesion.

## Visual Abstract

**Figure:**
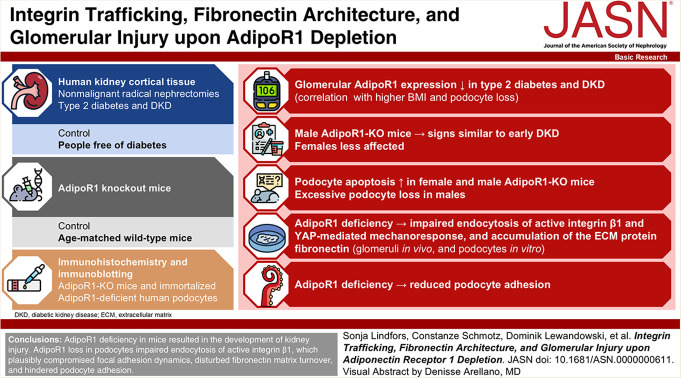

## Introduction

Obesity and type 2 diabetes are major risk factors for the development of CKD. Both conditions are associated with a decreased concentration of circulating adiponectin, an adipokine with anti-inflammatory, antifibrotic, and insulin-sensitizing properties.^1^ In rodents, adiponectin deficiency induces albuminuria,^2–4^ and administration of recombinant adiponectin ameliorates diabetic kidney disease.^2,5^ Particularly, glomerular podocytes, which maintain the integrity of the filtration barrier, are injured upon adiponectin deficiency.^2^ In humans, the contribution of adiponectin to the pathogenesis of diabetic kidney disease remains unclear. In individuals with obesity or type 2 diabetes without overt kidney disease, the lowered circulating adiponectin concentration is associated with elevated albuminuria or moderate kidney dysfunction.^6–10^ The contribution of hypoadiponectinemia to the progression of diabetic kidney disease was strengthened by a recent study that identified a protein-truncating mutation of the adiponectin gene that cosegregates with type 2 diabetes and diabetic kidney disease.^11^ On the other hand, circulating adiponectin concentration positively correlates with albuminuria in individuals with type 2 diabetes and lowered kidney function.^12,13^ This phenomenon, recognized as the adiponectin paradox, may result from adiponectin resistance, *i.e*., dysfunctional adiponectin signaling in the target tissue.^14^

Upon binding to adiponectin receptor 1 (AdipoR1) and adiponectin receptor 2, adiponectin promotes glucose uptake, lipid oxidation, and mitochondrial biogenesis through multiple signaling pathways.^1^ AdipoR1 is the predominant adiponectin receptor (AdipoR) in the kidney.^2,3,15,16^ Downstream AdipoR signaling is mediated by its intrinsic ceramidase activity,^1,17^ and binding of APPL1 (adapter protein, phosphotyrosine interacting with pleckstin homology domain and leucine zipper 1) to its intracellular *N*-terminus.^1^ The pathophysiological role of AdipoR1 has been studied in metabolic dysfunction,^18^ retinal degeneration,^19,20^ neurodegeneration,^21^ and acute myocardial infarction.^22^ Decreased expression of AdipoR1 in the kidney is observed in experimental diabetes models^16,23^ and human glomeruli in diabetic kidney disease^23^; however, it remains unknown whether AdipoR1 deficiency contributes directly to the development of kidney injury.

Podocyte adhesion to the glomerular basement membrane (GBM), by transmembrane integrins, is crucial for normal podocyte function and the integrity of the filtration barrier.^24^ Integrin *β*1, a component of integrin *α*3*β*1, is the primary integrin anchoring the podocyte to the GBM.^24,25^ Adhesion sites also serve as cellular mechanosensors, thereby controlling cell fate decisions, influencing the structural composition of the GBM, and remodeling the actin cytoskeleton.^24,26^ Because integrin alterations contribute to foot process effacement and podocyte loss,^27–30^ targeting integrin signaling is a promising strategy to prevent glomerular injury.^31–33^

We hypothesized that diminution of glomerular AdipoR1 predisposes to the development of kidney injury. Because APPL1 regulates endocytic cargo sorting^34^ and integrin *β*1 is among the most abundant endocytic cargo proteins in podocytes,^35^ we further hypothesized that AdipoR1 depletion may impair endocytic integrin trafficking in podocytes, thereby compromising normal podocyte–GBM interaction.

## Methods

### Human Kidney Tissue and Serum Samples

Human kidney cortical tissue was collected from the nonmalignant part of the radical nephrectomies performed at the Helsinki University Hospital, Finland, from people diagnosed with type 2 diabetes and type 2 diabetes–associated kidney disease (based on medical history records). The tissue was fixed in 10% formalin and embedded in paraffin using standard procedures. People free of diabetes served as controls. Except for people with diabetic kidney disease, individuals with decreased kidney function (eGFR <60 ml/min) were excluded from the analysis. Characteristics of the nephrectomized participants are summarized in Supplemental Table 1. Fasting blood samples were obtained from either lean people (body mass index [BMI] <25) or people with obesity (BMI >30). Characteristics of the participants are summarized in Supplemental Table 2. The use of human material was approved by the Ethical Committee of Helsinki University Hospital. Written informed consent was obtained from all study participants.

### AdipoR1 Knockout Mice

The mouse strain, B6.129P2-Adipor1tm1Dgen/Mmnc, research resource identifier: MMRRC_011599-UNC, was obtained from the Mutant Mouse Resource and Research Center at the University of North Carolina at Chapel Hill, a National Institutes of Health–funded strain repository. The strain was donated to the Mutant Mouse Resource and Research Center by Deltagen. The heterozygous mice were backcrossed into the C57BL/6J background. Homozygous AdipoR1 knockout (AdipoR1-KO) mice were created by interbreeding of the heterozygous mice. Age-matched wild-type (WT) controls were purchased from The Jackson Laboratory. Animals were housed under 12-hour light/12-hour dark cycles and bred using standard procedures. The study was approved by the Institutional Animal Care and Use Committee at University of California, Irvine, under Protocol AUP-21-096. All experimental procedures involving mice were performed according to the Guide for the Care and Use of Laboratory Animals published by the US National Institutes of Health.

At the age of 6 months (WT: 23.1±0.1 weeks; AdipoR1-KO: 24.0±0.1 weeks), the mice were euthanized by CO_2_ inhalation, followed by cervical dislocation. The dissected kidney samples were fixed in 10% formalin or embedded in Tissue-Tek optimal cutting temperature compound (Sakura Finetek, Torrance, CA) and snap-frozen, or they were prepared for electron microscopy as described below.

### Urine Analytics

Twenty-four–hour urine from 6-month-old AdipoR1-KO or WT mice was collected in individual metabolic cages, where the mice had free access to food and water. The urinary albumin and creatinine concentrations were measured at the Biochemical Analysis Core for Experimental Research (University of Helsinki).

### Immunohistochemistry and Immunocytochemistry

All antibodies and their dilutions are listed in Supplemental Table 3. Paraffin sections of human or mouse kidney (4 *μ*m) were prepared for immunohistochemistry using standard procedures. Human sections were stained using LabVision Autostainer 480 (Fremont, CA). Antigen retrieval from deparaffinized kidney sections was performed with EnVision FLEX Target Retrieval Solution (Dako, Agilent, Santa Clara), 10 mM Tris-1 mM EDTA (pH 9.0), or 10 mM citrate buffer (pH 6.0) in Decloaking Chamber NxGen (Biocare Medical, Pacheco, CA). Sections were incubated overnight with primary antibodies at 4°C. AdipoR1 antibody was validated using cellular lysates of AdipoR1 knockdown (AdipoR1-KD) podocytes (Supplemental Figure 1A) because a failure of several commercial AdipoR1 antibodies to recognize endogenous AdipoR1 has been reported.^20^ Detection was performed using a EnVision FLEX+ kit (Dako; for human sections), EnVision+ kit (Dako; for mouse sections), or BrightVision+ Anti-IgG (Poly-HRP) kit (ImmunoLogic, Amsterdam, The Netherlands; for mouse sections), along with 3,3′-diaminobenzidine (Dako).

Frozen sections of mouse kidney (5 *µ*m) or podocytes cultured on coverslips were fixed in 4% paraformaldehyde (PFA), permeabilized with 0.1% Triton X-100 in PBS, and incubated overnight 4°C with primary antibodies (see Supplemental Table 3). For immunofluorescence staining, coverslips or tissue sections were incubated with secondary antibodies and Hoechst 33342 for 1 hour and mounted in Prolong Diamond (Life Technologies, Carlsbad, CA).

For the mouse-on-mouse staining (synaptopodin), the primary antibody was complexed with a rabbit anti-mouse IgG Fab fragment (315-007-003, Jackson ImmunoResearch, Baltimore Pike, PA), followed by a blocking step with normal mouse serum (015-000-120; Jackson ImmunoResearch) as previously described.^36^ For all staining procedures, antibodies were diluted in REAL Antibody Diluent (Dako). Nonspecific staining was blocked with CAS-Block (Life Technologies, Frederick, MD) for 30 minutes before incubation with the primary antibodies. Tissue slides were counterstained with Mayer's Hematoxylin (Dako). Normal rabbit IgG (Invitrogen) or mouse IgG (Invitrogen) as used as negative controls. Tissue sections were imaged using a 3DHISTECH Panoramic 250 FLASH III digital slide scanner with 20× magnification at the Genome Biology Unit supported by Helsinki Institute of Life Science and the Faculty of Medicine, University of Helsinki, and Biocenter Finland. Positive staining area (brightfield images) or mean fluorescence intensity was quantified in anonymously selected 15–20 glomeruli/section with the Histoquant module of the QuantCenter software (version 2.0, 3DHISTECH, Budapest, Hungary) or QuPath software (version 0.4.3), using the intensity thresholder tool.^37^ The data on human podocyte number (the number of WT1-positive cells/glomerular cross-sectional area) were originally obtained in our previous study.^38^ Cells were imaged with a Zeiss Axio Imager Z2 upright epifluorescence wide-field microscope (Zeiss, Oberkochen, Germany) using a 20× objective or a LSM780 inverted confocal microscope (Zeiss) using 10× and 20× objectives. Three images were acquired for each coverslip. Image analysis was performed using Imaris software.

### Electron Microscopy

For transmission electron microscopy, 1 mm×1 mm cubes were cut from kidney cortex samples and fixed in 2.5% glutaraldehyde and 2.5% PFA in 100  mM phosphate buffer (pH 7.4) at room temperature for 3 hours, postfixed in 1% OsO4 in the same buffer for 1 hour, stained *en bloc* in 1% uranyl acetate in 10% ethanol for 1 hour, dehydrated in ethanol, and embedded in LX-112 resin. For imaging, thin sections of three mice per group, stained with uranyl acetate and lead citrate, were examined with a JEM-1400 Transmission Electron Microscope (Jeol, Tokyo, Japan) and imaged at a magnification of 8000× and 30,000× with an Olympus-SIS Morada digital camera (Olympus Soft Imaging Solutions GmbH, Münster, Germany). Images were analyzed using Fiji ImageJ software (National Institutes of Health, Bethesda, MD). The mean width of podocyte foot processes was determined from five glomeruli/mouse (8000× magnification), and the mean thickness of the GBM was determined from five to six glomeruli/mouse (300,00× magnification).

### Cell Culture

Immortalized human podocytes (AB8/13)^39^ were maintained as previously described.^40^ To induce differentiation, podocytes were thermoswitched to 37°C for 10–14 days. When indicated, cells were treated with 10 *µ*g/ml LPS (L2630, MilliporeSigma, Burlington, MA), 100 ng/ml recombinant human TNF*a* (H8916, MilliporeSigma), 10 *µ*M E64 (E3132, MilliporeSigma), or vehicle (water) for 24 hours. Alternatively, after a short wash with PBS, podocytes were treated for 48 hours with 10% serum from each participant (Supplemental Table 2), in the absence of FBS. Serum treatments were performed with two biological replicates in three independent experiments. Cells were lyzed in RIPA buffer (1% Igepal, 0.5% sodium deoxycholate, 0.1% SDS, 150 mm NaCl, 50 mM TRIS-HCl, pH 8.0) supplemented with protease and phosphatase inhibitors (1× EDTA-free cOmplete protease inhibitor cocktail [Roche, Mannheim, Germany], 50 mM NaF, 1 mM Na_3_VO_4_).

### Lentiviral Infections and Generation of AdipoR1-KD Podocytes

Human lentiviral pLKO1 vectors expressing AdipoR1 short hairpin RNAs (shRNAs) (shRNA-1: TRCN000063047 with a target sequence CCACTTCTATGGAGTCTCCAA; shRNA-2: TRCN0000063043 with a target sequence GCCCACCATGCACTTTACTAT; shRNA-3: TRCN0000063045 with a target sequence CGTCTATTGTCATTCAGAGAA) and empty pLKO1 vector with nontargeting scramble shRNA were obtained from the Functional Genomics Unit of University of Helsinki (Helsinki, Finland). Lentiviruses were produced by transfecting CMVDelta8.9 and phCMVg packaging plasmids, together with the pLKO1 construct, into HEK293FT cells (Invitrogen) with Lipofectamine 2000 (Invitrogen) (for details, see ref. 40). Transient transfection of shRNA was achieved by transducing podocytes on days 9–11 of differentiation. The virus-containing medium was replaced with a regular medium after 24 hours. To overcome the safety concerns of handling transiently transduced live cells during the live assays, podocyte cell lines stably expressing AdipoR1 shRNA-2 or AdipoR1 shRNA-3 or scramble shRNA (control) were obtained by selecting the shRNA-expressing proliferating podocytes with 1 *µ*g/ml puromycin (InvivoGen, San Diego, CA) for 9 days. In AdipoR1-KD podocytes, the expression of AdipoR1 protein was decreased by an average of 68% (shRNA-1), 79% (shRNA-2), and 60% (shRNA-3), compared with scramble shRNA-transduced podocytes (Supplemental Figure 1, B–G).

### Immunoblotting

Cellular or kidney tissue lysates with equal amounts of protein were separated on SDS-PAGE. Before loading, samples were supplemented with Laemmli sample buffer and incubated at 37°C for 10 minutes (podocyte lysates)^20^ or at 98°C for 5 minutes (kidney lysates). Integrin *β*1 was probed under nonreducing conditions in the absence of *β*-mercaptoethanol. Immunoblotting was performed as previously described.^40^ Antibodies used are presented in Supplemental Table 3.

### Apoptosis Assay

The level of apoptosis was measured as described^40^ by double staining for AnnexinV-FITC and 7-Aminoactinomycin D (BD Biosciences, Franklin Lakes, NJ). After the staining, cells were fixed in 4% PFA for 10 minutes and washed once with PBS. A minimum of 1.5×10^4^ cells were counted by a BD Accuri C6 flow cytometer (BD Biosciences). Cells positive for AnnexinV-FITC but negative for 7-Aminoactinomycin D were defined as apoptotic.

### Cytokine Assays

Secreted cytokines were measured with Q-Plex cytokine assays (Quansys Biosciences, Logan, UT). Imaging was performed using a Q-View Imager LS camera (Quansys Biosciences), and data were analyzed using Q-View software (Quansys Biosciences). The cytokine concentration in the culture medium (pg/ml) was normalized to the protein concentration (*µ*g/ml) of the cellular lysate prepared after collection of the medium. The experiment was performed with 6–7 biological replicates/treatment.

### Integrin *β*1 Endocytosis Assay

Differentiated scramble and AdipoR1-KD podocytes grown on coverslips were surface stained for active integrin *β*1^41^ (dilution 1/80) in FBS-free RPMI-1640 for 15 minutes on ice. After a quick wash with PBS, endocytosis was initiated by incubating the cells in a RPMI-1640 medium containing 2% FBS in a cell incubator at 37°C for the indicated time points. After the incubation, the cells were quickly washed with PBS and fixed in 4% PFA for 10 minutes. For the 0-minute time point, podocytes were surface stained for active integrin *β*1 for 15 minutes on ice, washed with PBS, and fixed. Permeabilization, immunostaining for Rab5, and secondary antibody incubations were performed as described below.

The quantification of the number of active integrin *β*1–positive vesicles was performed using the spot module of Imaris software (version 9.9.1, Oxford Instruments, Abingdon, United Kingdom) and was based on the size and local intensity of the vesicles positive for active integrin *β*1. The number of vesicles was determined for each time point of the endocytosis assay, with the 0-minute time point defined as the baseline. The number of vesicles at the subsequent time points is presented as fold-change over the baseline. Rab5-positive vesicles were detected similarly, and colocalization was assessed using the Spot-to-spot analysis tool of Imaris software (Oxford Instruments).

### Fluid Flow Shear Stress Assay

Fluid flow shear stress (FFSS) experiments were performed using the vitro.alive 2D integrated fluid flow chamber system for live cell microscopy from Vitroscope AS (Trondheim, Norway), combined with a peristaltic pump (BQ80S Microflow Variable-Speed, with DW10-3 pump head, Golander LLC, Norcoss, GA) to apply controlled fluid flow on the cell monolayer. AdipoR1-KD or scramble podocytes were seeded on glass slides and differentiated for 10–14 days. Thereafter, the glass slides were placed into the flow chamber in the vitro.alive device and exposed to FFSS of 2 dyne/cm^2^ for 2 hours in a normal culture medium under temperature-controlled (37°C) conditions. Live-cell recording was performed using a Nikon D5100 camera (lens: AF-S DX Nikkor 18–55 mm f/3.5–5.6 G VR) integrated with an Olympus IX70 inverted brightfield microscope (20× objective; Olympus Soft Imaging Solutions GmbH). At the end, the cells were briefly washed with PBS and fixed with 2% PFA. Immunofluorescence staining was performed as described below. Independent experiments were performed six times per each cell line.

For measuring the median traveled distance per hour of the cells (vector length), the obtained live-cell recordings were parsed into images by using a custom Python script (version 3.12.3) that uses OpenCV library (version 4.9.0.80). Images were 120 seconds apart and in range of 60 minutes. The podocytes in the images were annotated using Computer Vision Annotation Tool (CVAT) software (version 2.15.0) and exported in Segmentation mask 1.1 format. The mask was imported to another custom Python script (version 3.12.3) that uses OpenCV (version 4.9.0.80), NumPy (version 1.26.4), and Pandas (version 2.2.2) libraries to calculate the centroid of each cell. In the mask, every cell had a unique color, and the script first counted all of the unique colors in the mask, followed by a binary representation of the mask for one color at a time. The centroid of the cell was calculated by using moments on the binary mask, and the data were saved to an Excel file. In Excel (Microsoft 365, version 2308), the movement of each cell was calculated by using the centroid data to get the median distance the cells travel in the video recordings.

### Cell-Derived Matrix Adhesion Assay

Cell-derived matrix plates were prepared as previously described.^42^ Briefly, AdipoR1-KD or scramble podocytes were differentiated on 24-well plates. For the last 7–10 days, the medium was supplemented with 50 *μ*g/ml L of ascorbic acid (Merck, Aubonne, Switzerland). The cell-derived matrix plates were decellularized by applying prewarmed (37°C) extraction buffer (0.5% Triton X-100/20 mM NH_4_OH in PBS) for 3 minutes at room temperature, followed by washing twice with PBS. The residual DNA was eliminated by applying 15 U/ml DNase I (in PBS supplemented with 1 mM CaCl_2_ and 1 mM MgCl_2_) for 30 minutes at 37°C, followed by three washes with PBS. Next, differentiated scramble podocytes grown on 10-cm plates were seeded on the prepared cell-derived matrix plates and allowed to adhere for 2 hours at 37°C. This was followed by several washes with PBS, fixing with 4% PFA, and staining with 0.1% crystal violet in 2% ethanol for 1 hour. After several washes with PBS over 5 hours, the plate was imaged with an Odyssey Infrared Imaging System (LI-COR, Bad Homburg von der Höhe, Germany), and the data were analyzed using Image Studio software (LI-COR). The experiment was repeated four times with 7–9 biological replicates in each experiment.

### Transferrin Uptake Assay

Differentiated AdipoR1-KD or scramble podocytes were starved in FBS-free media for 1 hour and subsequently incubated with 25 *µ*g/ml Alexa Fluor 594–conjugated human transferrin (T13343 Invitrogen) in a complete medium (without insulin-transferrin-selenium supplement) for 15 or 30 minutes. After placing the plate on ice and rinsing the cells with cold PBS, the cell surface was acid stripped with cold 1% acetic acid in 0.5 M NaCl for 30 seconds. The cells were fixed with 4% PFA, stained with DRAQ5, and imaged with the Odyssey Infrared Imaging System (LI-COR). The experiment was repeated three times with 48 biological replicates per cell line in each experiment.

### Statistical Analyses

Data are presented as mean±SD unless otherwise indicated. Statistical analyses were performed using Graphpad Prism software (version 9.2.0., San Diego, CA). To compare means between two groups, unpaired two-tailed Student's *t* test was performed. To compare means between three groups, one-way ANOVA with Bonferroni *post hoc* test was performed. To compare means between two groups with several time points, two-way ANOVA with Bonferroni *post hoc* test was performed. Bivariate correlations were analyzed with Pearson's test. *P* < 0.05 was considered statistically significant. *In vitro* experiments included 3–6 independent experiments with 3–9 biological replicates/treatment unless otherwise mentioned. Each scatter dot represents the data obtained from one mouse, one individual, or one well in a multiwell plate or one imaged area on the coverslip (representing on average 40 cells) unless otherwise mentioned.

## Results

### AdipoR1 Expression Was Decreased in the Glomeruli of People with Type 2 Diabetes and Correlated with Podocyte Number

**Figure 1:**
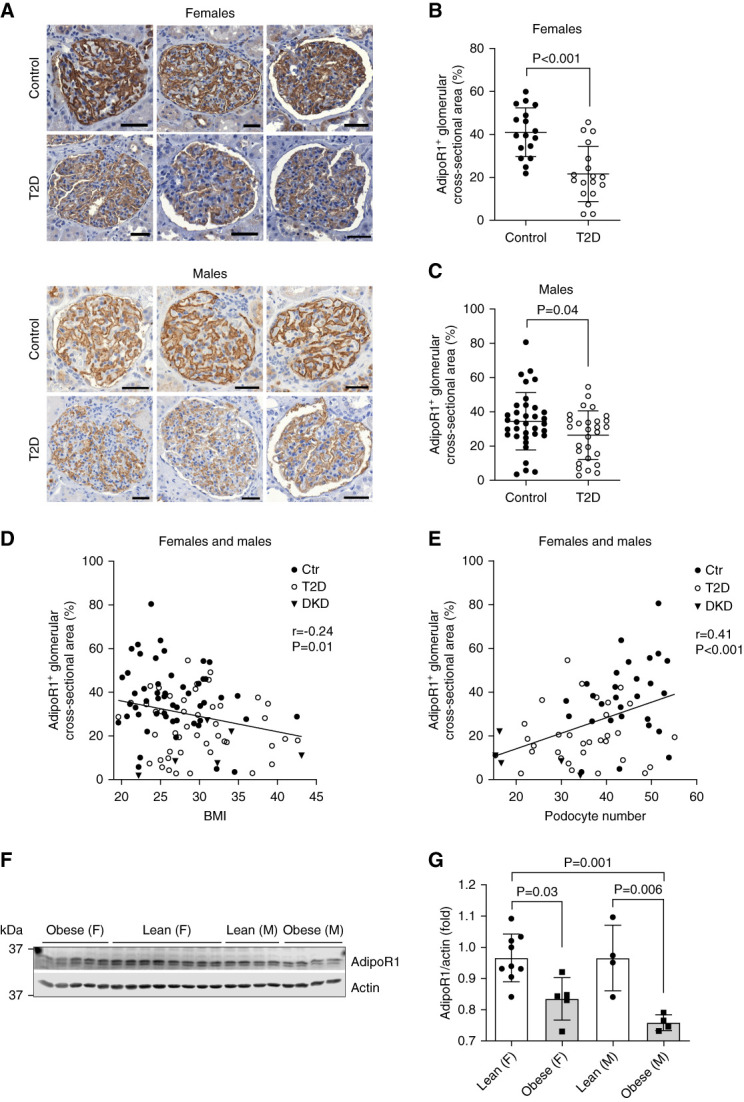
**Glomerular expression of AdipoR1 was decreased in people with type 2 diabetes and correlated with the podocyte number.** (A) Representative images of kidney sections immunohistochemically stained for AdipoR1, from women and men with type 2 diabetes without kidney complication (T2D) or people without diabetes (control). Scale bar: 50 *µ*m. (B and C) Quantification of glomerular AdipoR1-positive area in women (B) and in men (C) revealed that AdipoR1 expression was decreased in people with type 2 diabetes (T2D) compared with people without diabetes. Control women, *n*=17; women with T2D, *n*=18; control men, *n*=35; men with T2D, *n*=28. Data were assessed by two-tailed Student's *t* test. (D and E) Bivariate Pearson's correlation analysis identified a negative correlation between the glomerular AdipoR1-positive area and the BMI of study participants (D), and a positive correlation between the glomerular AdipoR1-positive area and the podocyte number (the number of WT1-positive cells within glomerular cross-sectional area) in control individuals, patients with T2D, and patients with DKD pooled together (E). In (D): *N*=104 (control women, *n*=17; women with T2D, *n*=18; control men, *n*=35; men with T2D, *n*=28; women with DKD, *n*=2; men with DKD, *n*=4). In (E): *n*=65 (control women, *n*=9; control men, *n*=19; women with T2D, *n*=15; men with T2D, *n*=17; women with DKD, *n*=2; men with DKD, *n*=3). The data for human podocyte number were originally observed in our previous study.^38^ (F) Representative immunoblots for AdipoR1 in cellular lysates of human podocytes treated for 48 hours with sera obtained from either lean or obese women (F) or men (M). (G) Quantification of AdipoR1 expression normalized to actin from immunoblots as in (F) revealed downregulation of AdipoR1 in podocytes treated with sera from people with obesity compared with lean people. Data were assessed by one-way ANOVA with Bonferroni correction. AdipoR1, adiponectin receptor 1; BMI, body mass index; Ctr, control; DKD, diabetic kidney disease; T2D, type 2 diabetes.

Immunohistochemistry in nephrectomized kidneys (*N*=104) revealed that AdipoR1 was strongly expressed in human glomeruli, especially in podocytes (Figure 1A). Glomerular expression of AdipoR1 was 48% lower in women (*n*=18) and 28% lower in men (*n*=28) (Figure 1, B and C) with type 2 diabetes without kidney complication compared with people without diabetes (women, *n*=17; men, *n*=35). Glomerular AdipoR1 expression was also decreased in people with type 2 diabetes–associated kidney disease compared with control people (Supplemental Figure 2, A and B). Correspondingly, AdipoR1 mRNA was downregulated in people with type 2 diabetes–associated kidney disease compared with control people in publicly available transcriptome data for isolated glomeruli^43^ (Supplemental Figure 2C). The glomerular expression of AdipoR1 correlated inversely with BMI of participants (Figure 1D) and positively with the podocyte number (Figure 1E). In addition, the expression of AdipoR1 was lower in immortalized podocytes exposed to sera obtained from obese individuals compared with treatments with sera obtained from lean people (Figure 1, F and G). Collectively, type 2 diabetes and obesity were associated with lower AdipoR1 expression in glomerular cells, potentially causing adiponectin resistance.

### AdipoR1 Depletion Led to the Development of Glomerular Injury in Mice

**Figure 2:**
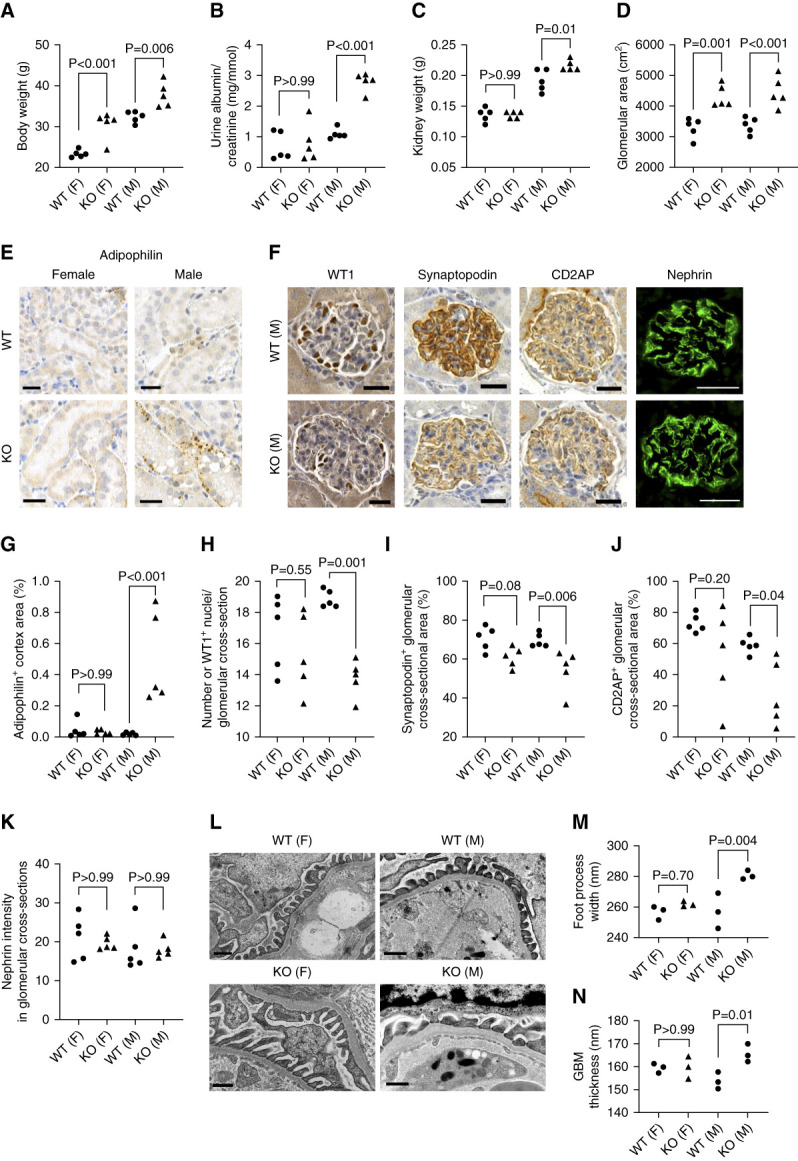
**AdipoR1-KO mice developed glomerular injury.** (A) Both female (F) and male (M) AdipoR1-KO mice weighed more than WT controls at the age of 6 months (*n*=5/group). (B) Albumin/creatinine levels were higher in urine from male AdipoR1-KO mice compared with WT controls, whereas no difference was observed for females (*n*=5/group). (C) Total kidney weight (g) was greater in male AdipoR1-KO mice but not in female AdipoR1-KO mice (*n*=5/group). (D) Glomerular cross-sectional area was greater in AdipoR1-KO mice (*n*=5/group). (E and F) Representative immunohistochemical or immunofluorescence staining of AdipoR1-KO and WT mouse kidney sections for adipophilin (marker of lipid droplets) (E), WT1 (nuclear podocyte marker) (F), CD2AP (F), synaptopodin (F), and nephrin (F). Scale bar: 25 *µ*m. (G) Quantification of adipophilin-positive kidney cortex area from images as in (E) revealed an accumulation of ectopic lipid droplets in male AdipoR1-KO mice (*n*=5/group). (H) Quantification of WT1-positive nuclei within glomerular cross-sections from images as in (F) revealed a decrease in podocyte number in male AdipoR1-KO mice compared with WT controls (*n*=5/group). (I and J) Quantification of synaptopodin- and CD2AP-positive glomerular area from images as in (F) showed decreased levels of both proteins in male AdipoR1-KO mice compared with WT controls (*n*=5/group). (K) Quantification of mean fluorescence intensity of nephrin in glomerular cross-sections from images as in (F) showed no difference in nephrin levels between AdpoR1-KO and WT mice (*n*=5/group). (L) Representative transmission electron microscopic images of male and female AdipoR1-KO and WT mouse kidneys. Scale bar: 500 nm. (M and N) Mean podocyte foot process width (M) and thickness of the GBM (N), quantified from images as in (L), were greater in male, but not in female, AdipoR1-KO mice compared with WT controls (*n*=3/group). (A–N) Data were assessed by Student's *t* test (two groups) or one-way ANOVA with Bonferroni correction (four groups). AdipoR1-KO, AdipoR1 knockout; GBM, glomerular basement membrane; KO, knockout; WT, wild-type.

At the age of 6 months, AdipoR1-KO mice had gained more weight than the WT controls (Figure 2A). The 24-h urinary albumin/creatinine level (Figure 2B), total kidney weight (Figure 2C), and ectopic lipid droplet accumulation in the kidney cortex (Figure 2, E and G) were elevated in male, but not female, AdipoR1-KO mice, whereas glomerular area was elevated in both females and males (Figure 2D). Podocyte number (Figure 2, F and H, and Supplemental Figure 3A) and the expression of the central podocyte proteins synaptopodin and CD2AP (Figure 2, F, I, and J) were decreased in male, but not female, AdipoR1-KO mice, while nephrin expression was unchanged (Figure 2, F and K). A larger glomerular area and lower expression of glomerular CD2AP, synaptopodin, and P-cadherin expression was already evident in 2.5-month-old male AdipoR1-KO mice, whereas no decrease in nephrin level or podocyte number was observed (Supplemental Figure 4).

Transmission electron microscopic analysis of 6-month-old AdipoR1-KO mice revealed that the mean width of podocyte foot processes and the mean thickness of the GBM were greater in males, but not females, compared with WT controls (Figure 2, L–N, and Supplemental Figure 3, B and C). Collectively, male AdipoR1-KO mice displayed structural and functional kidney abnormalities typical for diabetic kidney disease, whereas females were less susceptible to kidney injury.

### AdipoR1 Deficiency Induced Injury-Related Signaling and Caused Podocyte Hypertrophy and Apoptosis

**Figure 3:**
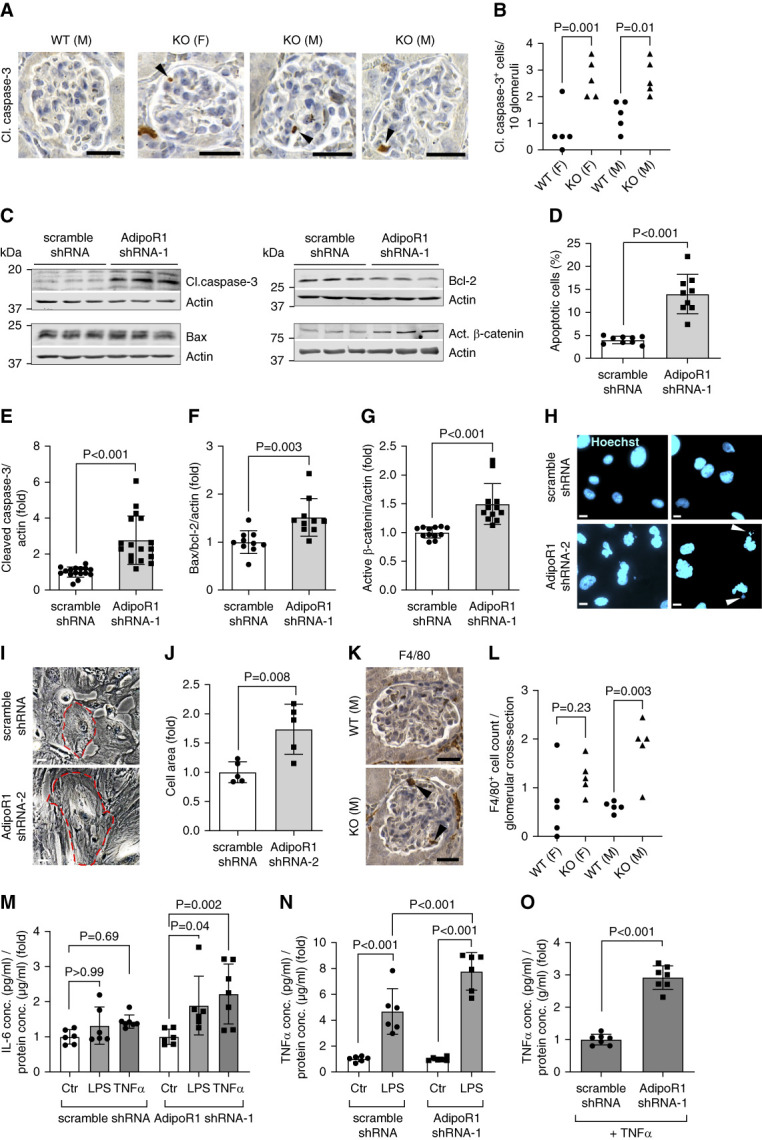
**AdipoR1 deficiency induced apoptosis and increased inflammation in immortalized podocytes and in the mouse glomeruli.** (A) Representative immunohistochemical staining of AdipoR1-KO and WT male (M) and female (F) mouse kidney sections for cleaved caspase-3 (apoptosis marker). Scale bar: 25 *µ*m. (B) Quantification of the number of cleaved caspase-3–positive cells per ten glomeruli (20 random glomeruli counted/mouse) revealed increased apoptosis of glomerular cells in female (F) and male (M) AdipoR1-KO mice compared with WT mice (*n*=5/group). (C) Representative immunoblots for cleaved caspase-3, Bax, Bcl-2, and active *β*-catenin in cellular lysates of transient AdipoR1-KD (by AdipoR1 shRNA-1) and control (scramble shRNA) podocytes. (D) The percentage of apoptotic AnnexinV-positive/7-AAD–negative podocytes measured by flow cytometry revealed an increase of apoptosis in transient AdipoR1-KD (by AdipoR1 shRNA-1) podocytes compared with control (scramble shRNA) podocytes. (E–G) The expression levels of cleaved caspase-3 (E), Bax to Bcl-2 ratio (F), and active *β*-catenin (G) normalized to actin were upregulated in transient AdipoR1-KD podocytes (by AdipoR1 shRNA-1) compared with control (scramble shRNA) podocytes quantified from immunoblots, as in (C). (H) Representative immunofluorescence images of Hoechst-stained nuclei showed an abnormal nuclear morphology and increased nuclear fragmentation (white arrowheads) in stable AdipoR1-KD podocytes (by AdipoR1 shRNA-2) compared with control (scramble shRNA) cells. Scale bar: 20 *µ*m. (I) Representative brightfield images for stable AdipoR1-KD (by AdipoR1 shRNA-2) and scramble (control) podocytes. Cell border is indicated in red. Scale bar: 50 *µ*m (J) Mean cell area (fold of control) was increased in stable AdipoR1-KD podocytes (by AdipoR1 shRNA-2). (K) Representative immunohistochemical staining of AdipoR1-KO and WT mouse kidney sections for F4/80 (macrophage marker). Scale bar: 25 *µ*m. (L) Quantification of F4/80-positive cells within glomerular cross-sections from images as in (K) showed increased infiltration of macrophages in male AdipoR1-KO mice compared with WT (*n*=5/group). (M) Secretion of IL-6 in response to treatments with LPS or TNF*α* for 24 hours was increased in transient AdipoR1-KD podocytes (by AdipoR1 shRNA-1), whereas no response was seen in control (scramble shRNA) podocytes. (N) Secretion of TNF*α* in response to treatment with LPS for 24 hours was increased in AdipoR1-KD podocytes (by AdipoR1 shRNA-1) compared with control (scramble shRNA) podocytes. (O) Secretion of TNF*α* (fold over control) in response to treatment with TNF*α* for 24 hours was increased in transient AdipoR1-KD podocytes (by AdipoR1 shRNA-1) compared with control (scramble shRNA) podocytes. Both endogenous and exogenous TNF*α* was measured in (O). (M–O) Data were visualized as a fold change of the cytokine secretion (pg/ml) normalized to the cellular protein concentration (*µ*g/ml) relative to the vehicle-treated cells. (B and L–N) Data were assessed by one-way ANOVA with Bonferroni correction. (D–G, I, and O) Data were assessed by two-tailed Student's *t* test. 7-AAD, 7-Aminoactinomycin D; AdipoR1-KD, AdipoR1 knockdown; shRNAs, short hairpin RNAs.

An increased number of cleaved caspase-3–positive apoptotic cells was observed in the glomeruli of AdipoR1-KO mice (Figure 3, A and B), similar amounts constituting podocytes in females and males (Supplemental Figure 3, D and E). An increase in apoptosis was also observed in transient AdipoR1-KD podocytes (AdipoR1 shRNA-1 or shRNA-3 transductions), visualized by an increase in AnnexinV-positive cells, caspase-3 cleavage, and the Bax–to–bcl-2 ratio (Figure 3, C–F, and Supplemental Figure 5, A and B). This was accompanied by decreased expression of CD2AP, synaptopodin, and P-cadherin (Supplemental Figure 6, A–D); attenuated insulin-mediated activation of the prosurvival Akt pathway (Supplemental Figure 6, G and H); and induction of the injury-related Wnt/*β*-catenin pathway (Figure 3, C and G) in transient AdipoR1-KD podocytes. In addition to nuclear fragmentation, a disruption of nuclear morphology, characteristic of mitotic catastrophe,^44,45^ was observed in stable AdipoR1-KD podocytes (Figure 3H). Morphologically, stable AdipoR1-KD podocytes were 1.7-fold larger than the control cells (Figure 3, I and J, and Supplemental Figure 5, C and D), indicating cell hypertrophy.

Because CD2AP and synaptopodin have been identified as direct targets of cytosolic cathepsin L–mediated proteolytic degradation, which promotes the development of glomerular injury,^46,47^ we evaluated whether cathepsin L activity contributes to the degradation of podocyte proteins upon AdipoR1 loss. By using CD2AP as an example, we found that treatment of transient AdipoR1-KD podocytes with E64, a cysteine cathepsin inhibitor that suppresses cytosolic cathepsin L activity,^46^ partially rescued the decrease in CD2AP (Supplemental Figure 6E). However, because the expression of CD2AP was downregulated also at the transcriptional level (Supplemental Figure 6F), cathepsin L–mediated degradation is not the solitary mechanism for CD2AP diminution. Collectively, loss of AdipoR1 decreased the expression of podocyte proteins and activated injury-related signaling, subsequently inducing apoptosis, which was also observed in the glomeruli of AdipoR1-KO mice.

### AdipoR1 Deficiency Increased Inflammatory Susceptibility in Immortalized Podocytes and Glomerular Infiltration of Macrophages in Mice

Because adiponectin mediates anti-inflammatory signaling,^40^ we evaluated whether AdipoR1 deficiency increases glomerular inflammation. Indeed, increased infiltration of macrophages was observed in the glomeruli of male AdipoR1-KO mice at the age of 2.5 and 6 months compared with WT mice (Figure 3, K and L, and Supplemental Figure 4, A and F). *In vitro*, transient knockdown of AdipoR1 aggravated the secretion of proinflammatory cytokines IL-6 and TNF*α* in response to LPS or TNF*α* stimulation compared with control podocytes (Figure 3, M–O). Collectively, AdipoR1 deficiency increases the inflammatory susceptibility of podocytes, which is a likely mechanism for increased glomerular infiltration of macrophages in AdipoR1-KO mice, further aggravating the inflammatory response.

### The Adapter Protein APPL1 Was Decreased in Type 2 Diabetes and upon AdipoR1 Loss

**Figure 4:**
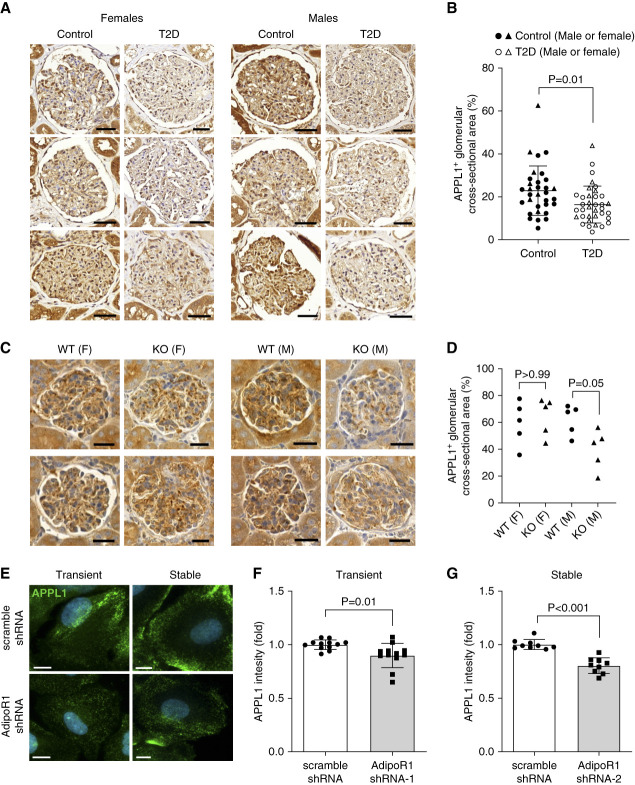
**APPL1 expression was decreased in the glomeruli of people with type 2 diabetes and AdipoR1-KO mice and in AdipoR1-KD podocytes.** (A) Representative images of immunohistochemical staining of kidney sections for APPL1 in women and men with type 2 diabetes without kidney complication (T2D) or people without diabetes (Control). Scale bar: 50 *µ*m. (B) Quantification of glomerular APPL1-positive area from images as in (A) revealed that APPL1 expression was decreased in patients with type 2 diabetes compared with people without diabetes. Control, *n*=32; T2D, *n*=36. (C) Representative immunohistochemical staining of female (F) and male (M) AdipoR1-KO and WT mouse kidney sections for APPL1. Scale bar: 25 *µ*m. (D) Quantification of APPL1-positive glomerular area from images as in (C) showed decreased levels of APPL1 in male (M) AdipoR1-KO mice compared with WT mice, whereas no difference was observed in females (F) (*n*=5/group). (E) Representative images of immunofluorescence staining for APPL1 in transient (by AdipoR1 shRNA-1) and stable (by AdipoR1 shRNA-2) AdipoR1-KD podocytes. Scale bar: 20 *μ*m. (F and G) Quantification of the mean fluorescence intensity of the APPL1 signal from images as in (E) revealed downregulation of APPL1 in both transient (F) and stable (G) AdipoR1-KD podocytes compared with control (scramble shRNA) cells. Hoechst-stained nuclei are indicated in blue. (B, F, and G) Data were assessed by two-tailed Student's *t* test. (D) Data were assessed by one-way ANOVA with Bonferroni correction.

The expression of APPL1, an intracellular signal mediator for AdipoR1,^1^ was decreased by 38% in the glomeruli of people with type 2 diabetes (*n*=36) compared with people without type 2 diabetes (*n*=32; Figure 4, A and B). A relatively small diminution of APPL1 was observed in the glomeruli of 6-month-old male, but not female, AdipoR1-KO mice (Figure 4, C and D). In immortalized podocytes, a reduction of APPL1 was seen upon transient and stable AdipoR1-KD (Figure 4, E–G). Collectively, deficiency of AdipoR1 is associated with a subsequent reduction of APPL1, except in female AdipoR1-KO mice.

### AdipoR1-KD Disturbed Endocytosis of Active Integrin *β*1 and Focal Adhesion Organization in Podocytes

**Figure 5:**
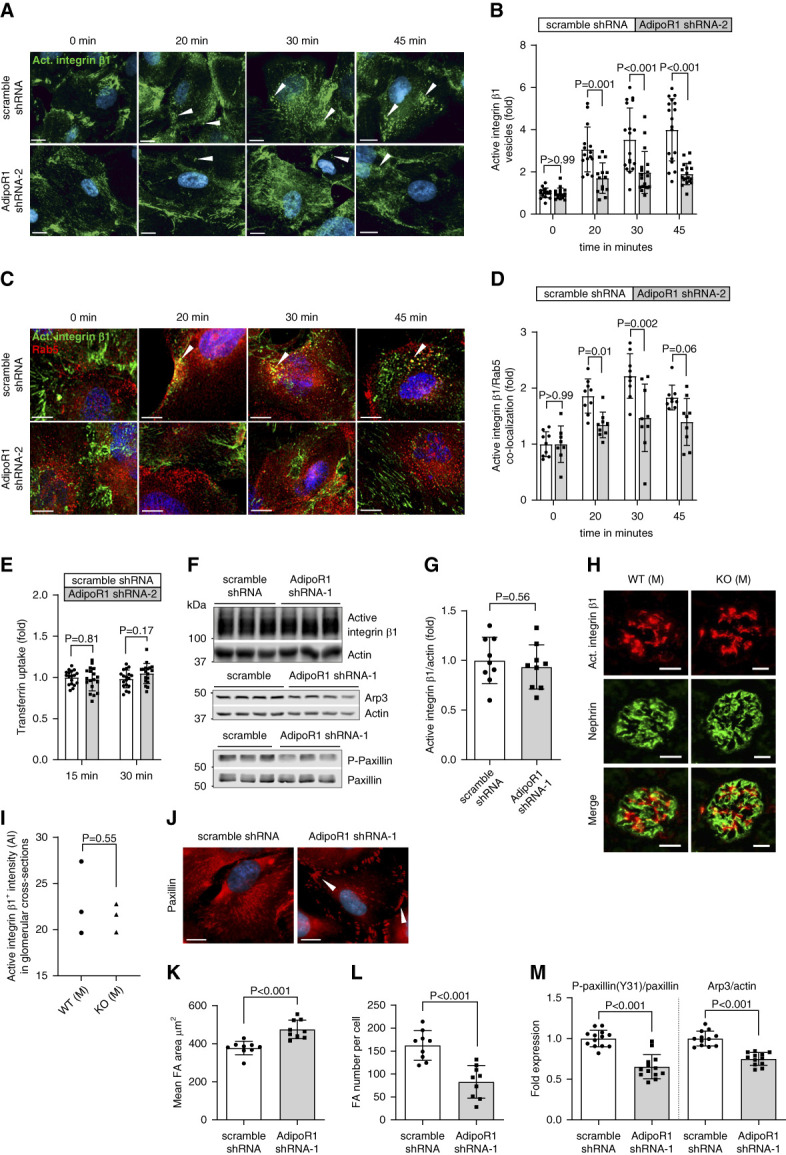
**AdipoR1-KD impaired active integrin *β*1 trafficking and focal adhesion turnover in podocytes.** (A) Representative immunofluorescence images of surface-labeled active integrin *β*1 at the plasma membrane (0-minute time point) and at 20-, 30-, and 45-minute time points after endocytosis (white arrowheads indicate vesicular structures) in stable AdipoR1-KD (by AdipoR1 shRNA-2) and control (scramble shRNA) podocytes. Hoechst-stained nuclei are indicated in blue. Scale bar: 20 *μ*m. (B) Quantification of active integrin *β*1–positive vesicular structures (fold change versus 0 minute) revealed a decrease in the endocytosis of active integrin *β*1 at 20-, 30-, and 45-minute time points in stable AdipoR1-KD podocytes (gray bars) compared with control (scramble shRNA) cells (white bars). (C) Representative confocal images of an immunofluorescence staining for Rab5 (red) and surface-labeled active integrin *β*1 (green) at 0-minute time point and after 20, 30, and 45 minutes of endocytosis in stable AdipoR1-KD podocytes. Colocalization of Rab5 and active integrin *β*1 is indicated with white arrowheads. Scale bar: 20 *μ*m. (D) Incidence of colocalization of surface-labeled active integrin *β*1 and Rab5 (fold change versus 0 minute) at 20-, 30-, and 45-minute time points was decreased in stable AdipoR1-KD podocytes (gray bars) compared with control (scramble shRNA) cells (white bars). (E) Quantification of the level of endocytosed transferrin at 15 and 30 minutes (normalized to DRAQ5) showed no difference between stable AdipoR1-KD and control (scramble shRNA) podocytes. Data are represented as fold over control (scramble shRNA) podocytes at 15 minutes. (F) Representative immunoblots for active integrin *β*1, Arp3, phosphorylated paxillin (P-Paxillin), and total paxillin in cellular lysates of transient AdipoR1-KD and control (scramble shRNA) podocytes. (G) The expression level of active integrin *β*1 normalized to actin was unchanged in AdipoR1-KD podocytes compared with control (scramble shRNA) podocytes quantified from immunoblots as in (F). (H) Representative immunofluorescence staining of AdipoR1-KO and WT male (M) mouse kidney sections for active integrin *β*1 (red) and nephrin (green; podocyte marker). Scale bar: 25 *μ*m. (I) Quantification of the mean fluorescence intensity of active integrin *β*1 within the glomeruli from images as in (H) showed no difference between male AdipoR1-KO and WT mice (*n*=3 mice/group; same animals as in Figure 2, L and M). (J) A representative immunofluorescence staining for paxillin (red), used to visualize the focal adhesions, in transient AdipoR1-KD (by AdipoR1 shRNA-1) and control (scramble shRNA) podocytes. Plaque-like appearance of focal adhesions is indicated with white arrowheads. Hoechst-stained nuclei are indicated in blue. Scale bar: 20 *μ*m. (K and L) Mean area of focal adhesions was increased (K), and total number of focal adhesions per cell decreased (L) in transient AdipoR1-KD (by AdipoR1 shRNA-1) podocytes compared with control (scramble shRNA) podocytes, quantified from images as in (J). (M) The ratio of phosphorylated paxillin (Y31) to total paxillin and the expression level of Arp3 (normalized to actin) were downregulated in transient AdipoR1-KD (by AdipoR1 shRNA-1) podocytes compared with control (scramble shRNA) podocytes quantified from immunoblots as in (F). (B and D) The quantification of vesicles positive for active integrin *β*1 or Rab5 was based on their local intensity and size. The number of vesicles was determined for each time point of the endocytosis assay, with the 0-minute time point defined as the baseline. Data were assessed by two-way ANOVA with Bonferroni correction. (E–M) Data were assessed by Student's *t* test. FA, focal adhesion.

APPL1, an effector protein for Rab5,^34^ is an early regulator of endocytic trafficking of integrins.^34,48^ We hypothesized that loss of AdipoR1–APPL1 signaling may alter integrin *β*1 endocytosis. Indeed, we observed that stable AdipoR1 deficiency correlated with diminished internalization of active (ligand-bound) integrin *β*1 at 20-, 30-, and 45-minute time points after the start of endocytosis (Figure 5, A and B, and Supplemental Figure 5, E and F). Furthermore, colocalization of active integrin *β*1 with Rab5 (expressed as fold-change relative to 0 minute), a marker of early endosomes that also regulates later stages of endocytic processes,^49^ was decreased at 20- and 30-minute time points in AdipoR1-KD podocytes, confirming decreased endocytosis of active integrin *β*1 (Figure 5, C and D, and Supplemental Figure 5, G and H). Endocytosis assay for labeled transferrin showed no difference between the cell lines ruling out the effect of AdipoR1 on clathrin-mediated endocytic processes in a global manner (Figure 5E). The total level of active integrin *β*1 was not altered in AdipoR1-KD podocytes (Figure 5, F and G) or AdipoR1-KO mice (Figure 5, H and I).

Because endocytosis of active integrin *β*1 reportedly affects focal adhesion organization and turnover,^50^ we postulated that these processes might be altered in AdipoR1-KD podocytes. Transient knockdown of AdipoR1 caused a plaque-like appearance of focal adhesions (visualized by immunostaining for paxillin) (Figure 5J), which resulted in a 26% increase in the mean focal adhesion area (Figure 5K) and 49% decrease in the total cellular number of focal adhesions (Figure 5L). The expression levels of Arp3 and tyrosine-phosphorylated paxillin (Y31), which control the maturation and turnover of focal adhesions,^51–53^ were decreased in AdipoR1-KD podocytes (Figure 5, F and M). Arp3 expression was unchanged in the kidneys of AdipoR1-KO mice (Supplemental Figures 3, F and G, and 4, G and J). Collectively, loss of AdipoR1, in association with decreased APPL1, impairs the endocytosis of active integrin *β*1 and disrupts focal adhesion organization and turnover.

### AdipoR1 Deficiency Impaired Podocyte Adhesion by Extracellular Matrix Remodeling

**Figure 6:**
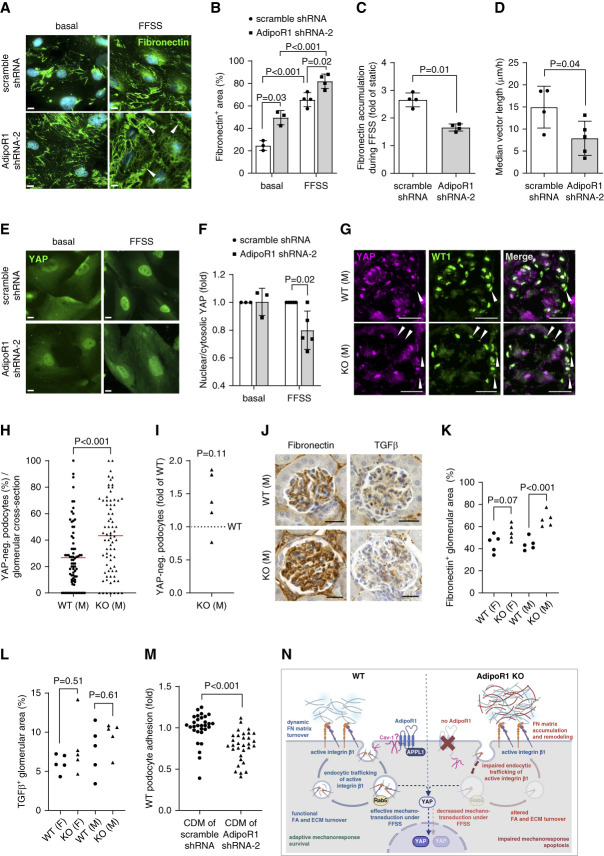
**AdipoR1 deficiency impaired podocyte mechanosensing and induced fibronectin accumulation.** (A) Representative immunofluorescence staining for fibronectin (green) under basal (normal cell culture) and FFSS conditions in stable AdipoR1-KD (by AdipoR1 shRNA-2) and control (scramble shRNA) podocytes. Nuclei were counterstained with Hoechst stain (blue). Arrowheads indicate increased webbed appearance of the fibronectin fibrils in the cell periphery. Scale bar: 20 *μ*m. (B) Quantification of the fibronectin-positive area from images as in (A) showed an upregulation of fibronectin expression in stable AdipoR1-KD podocytes (gray bars) compared with control (scramble shRNA) podocytes (white bars), both under basal and FFSS conditions. (C) Fold accumulation of fibronectin (as in B) in response to FFSS is weaker in AdipoR1-KD podocytes (gray bars) compared with control (scramble shRNA) cells (white bars). (D) Median vector length (*µ*m/h), representing the median distance traveled by AdipoR1-KD (gray bars) and control (scramble shRNA) podocytes (white bars), indicated that AdipoR1-KD podocytes migrate shorter distances than the control cells under FFSS exposure. (E) Representative immunofluorescence staining for YAP (green) under basal and FFSS conditions in stable AdipoR1-KD and control (scramble shRNA) podocytes. Scale bar: 20 *μ*m. (F) Quantification of the fluorescence signal for nuclear YAP relative to cytosolic YAP under basal and FFSS conditions from images as in (E) (fold over scramble shRNA) revealed impaired YAP activation, indicated by decreased nuclear translocation, in response to FFSS, in stable AdipoR1-KD knockdown (gray bars) compared with control (scramble shRNA) podocytes (white bars). (G) Representative immunofluorescence staining of AdipoR1-KO and WT male mouse kidney sections for YAP (pink) and WT1 (green; podocyte marker). YAP-negative podocytes are indicated by white arrowheads. Scale bar: 25 *μ*m. (H and I) Quantification of the percentage of YAP-negative podocytes (podocytes identified by WT1 staining) in male (M) AdipoR1-KO and WT mouse kidneys, quantified from images as in (G). The percentage of YAP-negative podocyte nuclei was increased in the pooled analysis of AdipoR1-KO glomeruli (each scatter dot presents one glomerulus) compared with WT glomeruli (H), whereas no statistical difference was found between the average values of each mouse (*n*=5/group, I). In the pooled analysis (H), average values of each individual glomerulus were pooled together within the group (15 glomeruli/mouse; n=5/group). For the analysis of fluorescence signal for YAP, WT and AdipoR1-KO mice were pair-matched on the basis of the background fluorescence intensity, and the analysis of each KO/WT pair was performed using the same threshold parameters. In (I), the percentage of YAP-positive podocytes in each AdipoR1-KO mouse was represented as normalized to its pair-matched WT control. (J) Representative immunohistochemical staining of AdipoR1-KO and WT mouse kidney sections for fibronectin and TGF*β*. Scale bar: 25 *µ*m. (K and L) Quantification of positive staining area from images as in (J) revealed an accumulation of fibronectin (K) in the glomeruli of male AdipoR1-KO mice compared with WT mice, while no difference was observed in TGF*β* expression in either females or males (L; *n*=5 mice/group). (M) Readhesion of WT podocytes onto decellularized CDMs derived from stable AdipoR1-KD podocytes was decreased compared with readhesion onto CDMs derived from control (scramble shRNA) podocytes. Each experiment had at least six biological replicates per cell line. (N) Schematic cartoon depicting the effects of AdipoR1 depletion on podocytes. In WT podocytes, effective endocytic trafficking of active integrin *β*1 ensures functional focal adhesion and ECM turnover. Under FFSS, WT podocytes show effective mechanotransduction. When AdipoR1 is depleted (AdipoR1-KO), the endocytosis of active integrin *β*1 is impaired, focal adhesion turnover is altered, ECM-protein fibronectin accumulates, and its organization is altered. Under FFSS, podocytes lacking AdipoR1 show impaired mechanoresponse. (B–M) Data were assessed by Student's *t* test (two groups) or one-way ANOVA with Bonferroni correction (four groups). CDM, cell-derived matrix; ECM, extracellular matrix; FFSS, fluid flow shear stress; FN, fibronectin; YAP, yes-associated protein.

Active integrin *β*1 can be endocytosed in a caveolin-1–dependent manner, thereby regulating fibronectin turnover.^54^ Because caveolin-1 directly interacts with AdipoR1,^55^ we asked whether AdipoR1 deficiency regulates podocyte-derived fibronectin deposition. Indeed, we found that the area covered by fibronectin was increased by two-fold in AdipoR1-KD podocytes compared with control cells in basal (regular cell culture) conditions (Figure 6, A and B, and Supplemental Figure 5, I and J). In addition, the structure of the fibronectin network was shifted from a scattered fibrillar appearance in control podocytes to a densely webbed fibrillar phenotype in AdipoR1-KD podocytes (Figure 6A).

Because the extracellular matrix (ECM) composition and recycling of active integrin *β*1 contribute to cellular force perception and mechanotransduction,^56^ we studied the response of AdipoR1-KD podocytes to mechanical stress, which typically increases in diabetic kidney disease because of glomerular hyperfiltration,^57^ thereby challenging the podocytes. Upon exposure to FFSS, both control and AdipoR1-KD podocytes increased the deposition of fibronectin (Figure 6, A and B), as previously reported.^58^ However, the fold increase of FFSS-induced fibronectin accumulation was smaller in AdipoR1-KD podocytes than in control cells (Figure 6C); yet, the overall percent fibronectin-covered area in AdipoR1-KD cells still exceeded that of control cells under FFSS (Figure 6B). Notably, in AdipoR1-KD podocytes, the density and webbed appearance of the fibronectin fibrils increased upon FFSS exposure, especially at the cell periphery (Figure 6A). In addition, quantitative analysis of cell movement in response to FFSS from live-cell recordings revealed that AdipoR1-KD podocytes migrated shorter distances compared with control cells (Figure 6D, Supplemental Video 1 showing control podocytes and Supplemental Video 2 showing AdipoR1-KD podocytes).

We observed that FFSS-induced nuclear translocation (activation) of the mechanotransducer yes-associated protein (YAP) was attenuated in AdipoR1-KD podocytes (Figure 6, E and F, and Supplemental Figure 7). Decreased nuclear YAP levels were also observed in the pooled analysis of the glomeruli of male AdipoR1-KO mice (Figure 6, G and H), although the difference on the basis of the average values of each mouse did not reach statistical significance (Figure 6, G and I). Glomerular accumulation of fibronectin was observed in AdipoR1-KO male, but not female, mice (Figure 6, J and K), and no difference was observed in the glomerular profibrotic cytokine TGF*β* levels (Figure 6, J and L). Glomerular expression of EPB41L5, which controls the incorporation of collagen IV and laminin into the GBM,^42^ was unchanged in AdipoR1-KO mice (Supplemental Figure 3, F and H). Finally, to confirm whether excessive accumulation of fibronectin upon AdipoR1-KD functionally affects podocyte adhesion, we performed a reseeding assay on cell-derived matrices (CDMs) originating either from AdipoR1-KD or from control podocytes. Indeed, WT podocytes plated on CDMs of AdipoR1-KD podocytes showed decreased adhesion compared with the adhesion to control CDMs (Figure 6M and Supplemental Figure 5K). Collectively, AdipoR1 deficiency induced glomerular fibronectin accumulation, independent of TGF*β*, and impaired podocyte mechanosensing and adhesion by remodeling the ECM protein fibronectin organization (Figure 6N).

## Discussion

Adiponectin resistance, arising from impaired AdipoR-mediated signaling, is a potent contributor to the adiponectin paradox, *i.e*., kidney disease progression despite normal or increased adiponectin serum concentration.^13,14^ In this study, we characterized the functional contribution of AdipoR1 loss to the development of glomerular injury.

We observed that in human kidney, AdipoR1 was predominantly expressed in the glomerular podocytes. We also found that decreased expression of glomerular AdipoR1 was associated with type 2 diabetes and diabetic kidney disease, supported by previous studies,^16,23^ and correlated with podocyte loss in type 2 diabetes. Obesity seems to be a driving factor for the decrease in AdipoR1 because AdipoR1 level in glomeruli was inversely correlated with BMI and was decreased in podocytes treated with sera from obese people.

Given that glomerular AdipoR1 was decreased in type 2 diabetes without overt kidney complication, our data suggested a causative role of AdipoR1 deficiency in initiating glomerular injury. This interpretation was supported by our finding that AdipoR1-KO mice, particularly males, showed typical signs of early diabetic kidney disease, while female AdipoR1-KO mice were resistant to injury. Sex-dependent differences in lipid metabolism and ectopic adipose tissue accumulation, independent of excess body weight, contribute to the increased susceptibility of males to kidney diseases.^59^ Indeed, unlike females, male AdipoR1-KO mice displayed increased kidney weight and an accumulation of ectopic lipid droplets in the kidney, together with a mild increase in albuminuria and ultrastructural changes in the glomeruli.

We found that APPL1, the downstream signaling mediator of AdipoR1, was decreased in the glomeruli of people with type 2 diabetes, in AdipoR1-KD podocytes, and in the glomeruli of AdipoR1-KO mice. APPL1 exhibits a protective role in immortalized podocytes^60^ and contributes to the beneficial effects of adiponectin.^61^ In addition, CD2AP, synaptopodin, and P-cadherin were decreased upon AdipoR1 depletion, which may contribute to foot process effacement and albuminuria.^62,63^ Collectively, as lack of AdipoR1 impaired kidney function in male mice, it is likely that diminution of AdipoR1 in glomeruli in type 2 diabetes contributes to podocyte injury and progression of kidney disease.

We identified a novel function for AdipoR1 as a regulator of integrin *β*1 trafficking—knockdown of AdipoR1 impaired endocytosis of active integrin *β*1. This apparently contributed to podocyte injury upon loss of AdipoR1 because a strict balance of integrin expression, localization, and function in podocytes is essential for the integrity of the filtration barrier and the plasticity of foot processes.^24,25,27–30,64,65^ Impaired endocytosis potentially ensued from a decrease in APPL1, which regulates the initiation and cargo selection of endocytosis.^34^ We also observed in AdipoR1-KD podocytes a plaque-like appearance of the focal adhesions, indicating their dysfunctional turnover.^26,51,56,66,67^ This interpretation was supported by the concomitant downregulation of Arp3 and phosphorylated paxillin (Y31), both controlling focal adhesion turnover and morphology.^25,51,53,68–70^ Focal adhesion complexes involving paxillin and Arp3 anchor integrins to the actin cytoskeleton and contribute to the plasticity of foot processes.^25,51,53,68–70^ Indeed, we observed that AdipoR1-KD podocytes had attenuated migration response under FFSS, thus proposing a crucial role for AdipoR1 in mediating mechanosensing in podocytes. Collectively, our study identified AdipoR1 as a novel regulator of the endocytosis of active integrin *β*1 and focal adhesion assembly, suggesting that lack of AdipoR1 disrupts the adaptive function of the podocyte foot processes.

One major consequence of AdipoR1 deficiency was the glomerular accumulation of fibronectin, independently of the profibrotic cytokine TGF*β*.^71^ Glomerular ECM is a dynamic structure undergoing continuous remodeling.^72^ In normal conditions, fibronectin is a minor constituent of the glomerular ECM but becomes the predominant fibrotic component in excess ECM deposition.^73^ Our data indicated that podocytes contribute to the accumulation of glomerular fibronectin upon AdipoR1 deficiency, subsequently compromising their adhesion to the ECM. Intriguingly, trafficking of active integrins plays a central role in the maintenance and organization of the ECM,^56,74^ and importantly, active integrin *β*1 has been identified as an endocytic receptor for fibronectin turnover by caveolin-1–dependent endocytosis.^54^ Caveolin-1, the main structural component of caveolae, interacts with both AdipoR1 and APPL1.^55,75^ We propose that pathological accumulation of fibronectin results from its altered turnover and assembly, mediated by impaired trafficking of active integrin *β*1 caused by AdipoR1 deficiency. This is supported by previous data in myofibroblasts describing the role of the endocytosis of active integrin *β*1 as a pivotal regulator of fibronectin turnover.^54^

Accumulation of fibronectin contributes to the thickening and stiffening of the GBM,^58,71,76^ thereby impairing mechanosensing and YAP activation.^77,78^ We observed that activation of YAP did not differ at the basal conditions between AdipoR1-KD and control podocytes despite fibronectin accumulation in AdipoR1-KD cells, indicating that an increase of fibronectin alone did not affect YAP activity. However, upon exposure to FFSS, YAP activation was decreased in AdipoR1-KD podocytes, indicating an impaired mechanoresponse, whereas control podocytes were able to sense the mechanical stimulus and activate YAP, showing functional mechanotransduction. Not just the amount of fibronectin but also its architecture and network appearance were altered in AdipoR1-KD podocytes. This apparently explains the impaired mechanoresponse because the folding of fibronectin, regulated by mechanical force, affects its binding to integrin, and thereby, the degree of fibronectin folding adjusts outside-in mechanosignaling.^79,80^ The increase in fibrillar density and accumulation of the fibronectin matrix in the cell periphery after FFSS exposure in AdipoR1-KD podocytes further indicated disruption of the fibronectin matrix turnover.

Although fibronectin is essential for podocyte adhesion and cell spreading, especially during adaptation to mechanical stress,^58^ podocytes disfavor binding to fibronectin,^81^ which may explain their decreased adhesion to the CDMs derived from AdipoR1-KD podocytes. Furthermore, nuclear exclusion of YAP induces podocyte apoptosis and is seen in glomerular diseases, including diabetic kidney disease.^82–84^ Also synaptopodin and CD2AP levels were decreased upon AdipoR1 depletion, and interestingly, the failure of YAP translocation is mechanistically associated with the induction of cytosolic cathepsin L, leading to proteolytic degradation of synaptopodin and CD2AP^46,85^ that may further enhance podocyte loss. This supports our observations upon AdipoR1 depletion. Under glomerular hyperfiltration, the magnitude of FFSS is increased, enhancing podocyte detachment.^57^ Relating to our data, it is tempting to speculate that enhancing adiponectin signaling or preventing AdipoR1 loss protects from podocyte detachment in diabetic kidney disease by maintaining the effective turnover of the fibronectin matrix. A cartoon depicting the effects of lack of AdipoR1 is presented in Figure 6N.

Glomerular infiltration of macrophages increased in AdipoR1-KO mice, potentially because of the accumulation of excess ECM, known to facilitate macrophage infiltration in diabetic kidney disease.^86^ Furthermore, AdipoR1-KD in podocytes increased the magnitude of proinflammatory cytokine secretion upon inflammatory stimulation. Proinflammatory cytokines, including TNF*α* and IL-6, are upregulated in the glomeruli in diabetic kidney disease,^87^ amplify the inflammatory response by facilitating macrophage infiltration,^88^ and promote podocyte injury and apoptosis.^89^ Taken together, AdipoR1 deficiency presumably contributes to the chronic inflammatory status and increased podocyte loss in type 2 diabetes and is a potent target to alleviate chronic kidney inflammation, as our previous study also suggests.^40^

We found increased amount of cleaved caspase-3–positive apoptotic cells, also podocytes, in AdipoR1-KO glomeruli. Increased apoptosis, together with abnormal nuclear morphology that is a typical sign of mitotic catastrophe occurring because of the inability of podocytes to complete mitosis,^44,45^ was confirmed in immortalized AdipoR1-KD podocytes. Podocyte loss, whether through apoptosis, detachment, or mitotic catastrophe, is a major driver of diabetic kidney disease progression.^44,90^ Impaired integrin trafficking, decreased CD2AP and synaptopodin levels, failure to translocate YAP, increased inflammation, attenuated prosurvival Akt signaling, and activated *β*-catenin signaling, as shown in this study, are likely contributors to the podocyte apoptosis upon AdipoR1 deficiency.^46,91–94^ Likewise, previously reported ceramide accumulation and altered plasma membrane properties also are plausibly involved.^19,95–97^ Excessive podocyte loss in male AdipoR1-KO mice may result from failure to adhere to fibronectin-rich ECM, leading to detachment. In conclusion, podocyte apoptosis and detachment presumably contribute to the loss of podocytes in AdipoR1-KO mice.

Collectively, we found that AdipoR1 deficiency directly caused podocyte injury *in vitro* and *in vivo* and was associated with podocyte loss in type 2 diabetes. Deficient AdipoR-mediated signaling has previously been suggested to explain the paradoxical association of high circulating adiponectin concentration with increased mortality and higher risk of kidney complications^14,98^ and is a potential contributor to the pathogenesis of type 2 diabetes and numerous other diseases.^99–101^ This study indicates that besides adiponectin deficiency,^2^ adiponectin resistance is a potent contributor to the initiation and progression of glomerular injury in diseases associated with AdipoR1 downregulation, including type 2 diabetes.

## Supplementary Material

## Data Availability

All data generated and analyzed during this study are available from the corresponding author upon reasonable request.

## Supplemental Material

Supplemental Video 1. Control podocytes under FFSS. The video shows extracted frames from 1-hour live-cell recordings of control podocytes exposed to 2 dyne/cm^2^ FFSS.

Supplemental Video 2. AdipoR1-KD podocytes under FFSS. The video shows extracted frames from 1-hour live-cell recording of AdipoR1-KD podocytes exposed to 2 dyne/cm^2^ FFSS.

Supplemental Figure 1. Antibody validation for AdipoR1 immunohistochemical staining and AdipoR1-KD efficiency in AdipoR1-KD podocytes.

Supplemental Figure 2. Glomerular expression of AdipoR1 protein and mRNA was decreased in people with type 2 diabetes who have diabetic kidney disease compared with control people.

Supplemental Figure 3. Podocyte loss and ultrastructural alterations of the glomeruli were evident in 6-month-old male AdipoR1-KO mice, despite no change in glomerular Arp3 and EPB41L5 expression levels.

Supplemental Figure 4. AdipoR1-KO mice showed increased glomerular area and decreased expression of podocyte proteins at the age of 2.5 months.

Supplemental Figure 5. Increased apoptosis and cell area, impaired active integrin *β*1 trafficking, accumulation of fibronectin, and reduced adhesion to CDMs in AdipoR1-KD podocytes created using shRNA-3.

Supplemental Figure 6. Expression of podocyte proteins and insulin-stimulated Akt response were decreased in AdipoR1-KD podocytes.

Supplemental Figure 7. AdipoR1-KD podocytes showed impaired nuclear translocation of YAP in response to FFSS.

Supplemental Table 1. Basic characteristics of the study participants who underwent radical nephrectomy.

Supplemental Table 2. Basic characteristics of the study participants whose sera were used in the treatments of immortalized human podocytes.

Supplemental Table 3. Primary and secondary antibodies used in the study.
