# Supplementary material for: Integrin Trafficking, Fibronectin Architecture, and Glomerular Injury upon Adiponectin Receptor 1 Depletion
Source: J Am Soc Nephrol. 2025 Jan 28;36(5):825–44. doi: 10.1681/ASN.0000000611 (PMC12059104; doi:10.1681/ASN.0000000611)
Supplement: Supplementary file 1 [file jasn-36-825-s001.pdf]

Online supplemental material for:

Integrin trafficking, fibronectin architecture, and glomerular injury upon adiponectin receptor 1 depletion

Sonja Lindfors, PhD, Constanze Schmotz, PhD, Dominik Lewandowski, PhD, Annika Hau, MSc, Leena Saikko, Eero Lehtonen, MD, PhD, Ville Majaniemi, Minna Karhe, BSc, Jette-Britt Naams, MSc[1], Harry Nisen, MD, PhD, Jukka Tienari, MD, PhD, Moin A Saleem, FRCP, PhD, Prof, Katharina Pfeil, Dipl.Biol, Heiko Bugger, MD, PhD, Prof , Kirsi H. Pietiläinen, MD, PhD, Prof , Tuomas Mirtti, MD, PhD, Krzysztof Palczewski, PhD, Prof, Sanna Lehtonen, PhD, Prof

Supplemental Figure 1. Antibody validation for AdipoR1 immunohistochemical staining, and AdipoR1 knockdown efficiency in AdipoR1 knockdown podocytes.

Supplemental Figure 2. Glomerular expression of AdipoR1 protein and mRNA were decreased in people with type 2 diabetes who have diabetic kidney disease compared to control people.

Supplemental Figure 3. Podocyte loss and glomerular ultrastructural alterations were evident in 6-month-old male AdipoR1-KO mice, despite of no change in glomerular Arp3 and EPB41L5 expression.

Supplemental Figure 4. AdipoR1-KO mice showed increased glomerular area and decreased expression of podocyte proteins at the age of 2.5 months.

Supplemental Figure 5. Increased apoptosis and cell area, impaired active integrin β1 trafficking, accumulation of fibronectin, and reduced adhesion to cell-derived matrices in AdipoR1-KD podocytes created using shRNA-3.

Supplemental Figure 6. Expression of podocyte proteins and insulin-stimulated Akt response were decreased in AdipoR1 knockdown podocytes.

Supplemental Figure 7. AdipoR1 knockdown podocytes showed impaired nuclear translocation of YAP in response to fluid flow shear stress.

Supplemental Table 1. Basic characteristics of the study participants who underwent radical nephrectomy.

Supplemental Table 2. Basic characteristics of the study participants whose sera were used in the treatments of immortalized human podocytes.

Supplemental Table 3. Primary and secondary antibodies used in the study.

Supplemental Methods

Supplemental Video 1. Control podocytes under FFSS. The video shows extracted frames from one hour live-cell recordings of control podocytes exposed to 2 dyne/cm2 FFSS.
Supplemental Video 2. AdipoR1-KD podocytes under FFSS. The video shows extracted frames from one hour live-cells recording of AdipoR1-KD podocytes exposed to 2 dyne/cm2 FFSS.

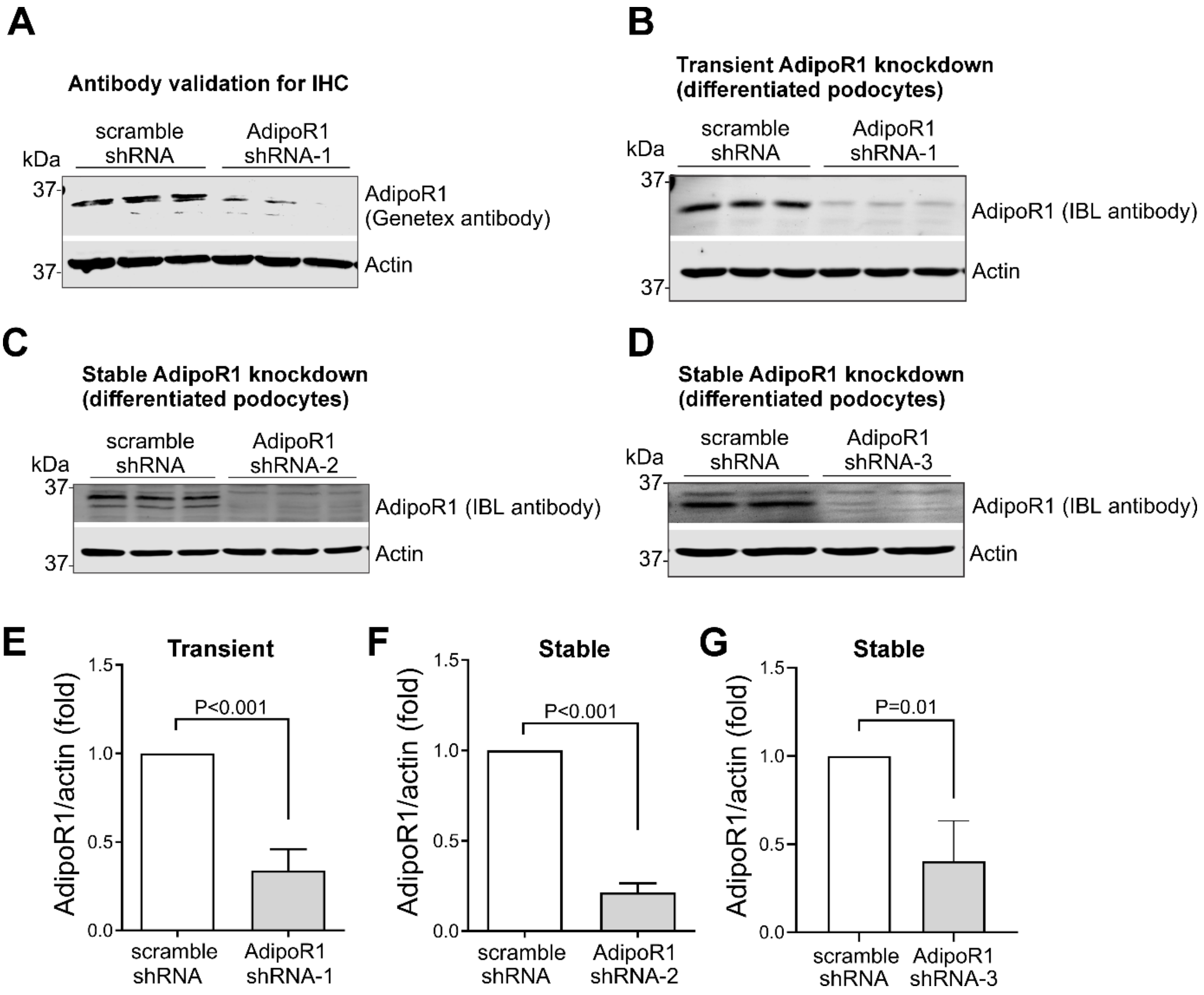

Supplemental Figure 1. Antibody validation for AdipoR1 immunohistochemical staining, and AdipoR1 knockdown efficiency in AdipoR1 knockdown podocytes. (A) Validation of the Genetex AdipoR1 antibody used for immunohistochemical (IHC) staining of human kidneys. Cellular lysates of human podocytes transiently transduced with scramble shRNA or AdipoR1 shRNA-1 were probed with the Genetex antibody. The antibody recognized a band at the expected size and the signal was decreased in the AdipoR1 knockdown podocytes confirming the specificity of the antibody. (B) Representative immunoblot for AdipoR1 (IBL antibody) in cellular lysates of human podocytes transiently transduced with scramble shRNA or AdipoR1 shRNA-1. (C,D) Representative immunoblots for AdipoR1 (IBL antibody) in cellular lysates of human podocytes with stable AdipoR1 knockdown mediated by shRNA-2 (C) or shRNA-3 (D) and respective control (scramble shRNA) cells. (E–G) Mean expression level of AdipoR1, normalized to actin, quantified from blots as in B–D, in cellular lysates of human podocytes with AdipoR1 knockdown achieved by either transient transduction of AdipoR1 shRNA-1 (E), or by stable selection after transducing proliferating podocytes with either AdipoR1 shRNA-2 (F) or AdipoR1 shRNA-3 (G).

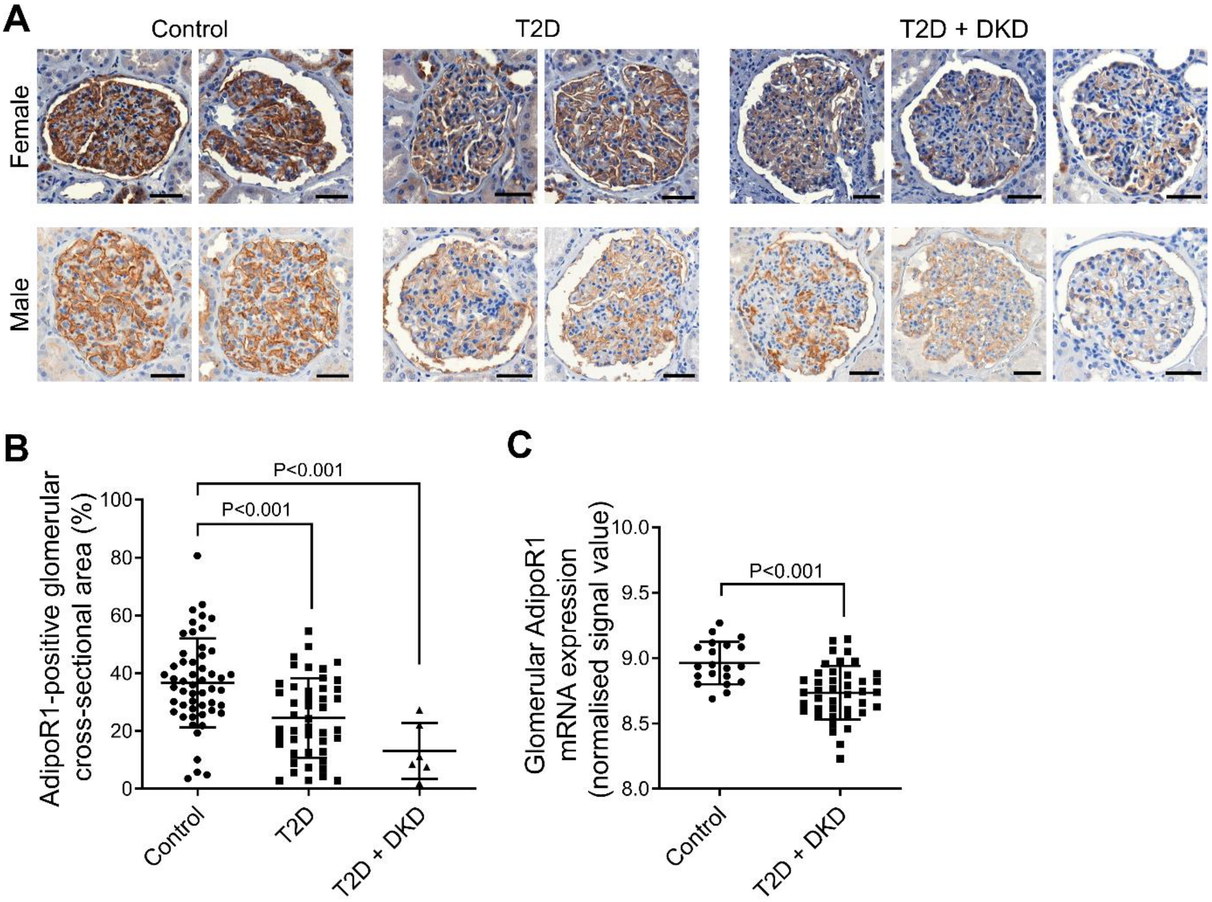

Supplemental Figure 2. Glomerular expression of AdipoR1 protein and mRNA were decreased in people with type 2 diabetes who have diabetic kidney disease compared to control people. (A) Representative images of immunohistochemical staining of kidney sections for AdipoR1 in females and males with type 2 diabetes without kidney complication (T2D), T2D and diabetic kidney disease (DKD), or people without diabetes (Control). Scale bar: 50 µm. (B) Quantification of images as in (A) revealed downregulation of AdipoR1 protein level in both people with T2D, and in people with T2D and DKD, compared to people without diabetes (Control). Control and T2D people are the same as in Figure 1, with images of kidneys of other individuals shown here. Here, females and males are combined. Data were assessed by one-way ANOVA with Bonferroni correction. Control, n=52; T2D, n=46; T2D with DKD, n=6. (C) Quantification of AdipoR1 mRNA expression in isolated glomeruli in a publicly available transcriptome database[1] revealed its downregulation in people with T2D and DKD in comparison to control people without diabetes (Control). Data were assessed by Student's t-test. Control, n=20; T2D and DKD, n=41.

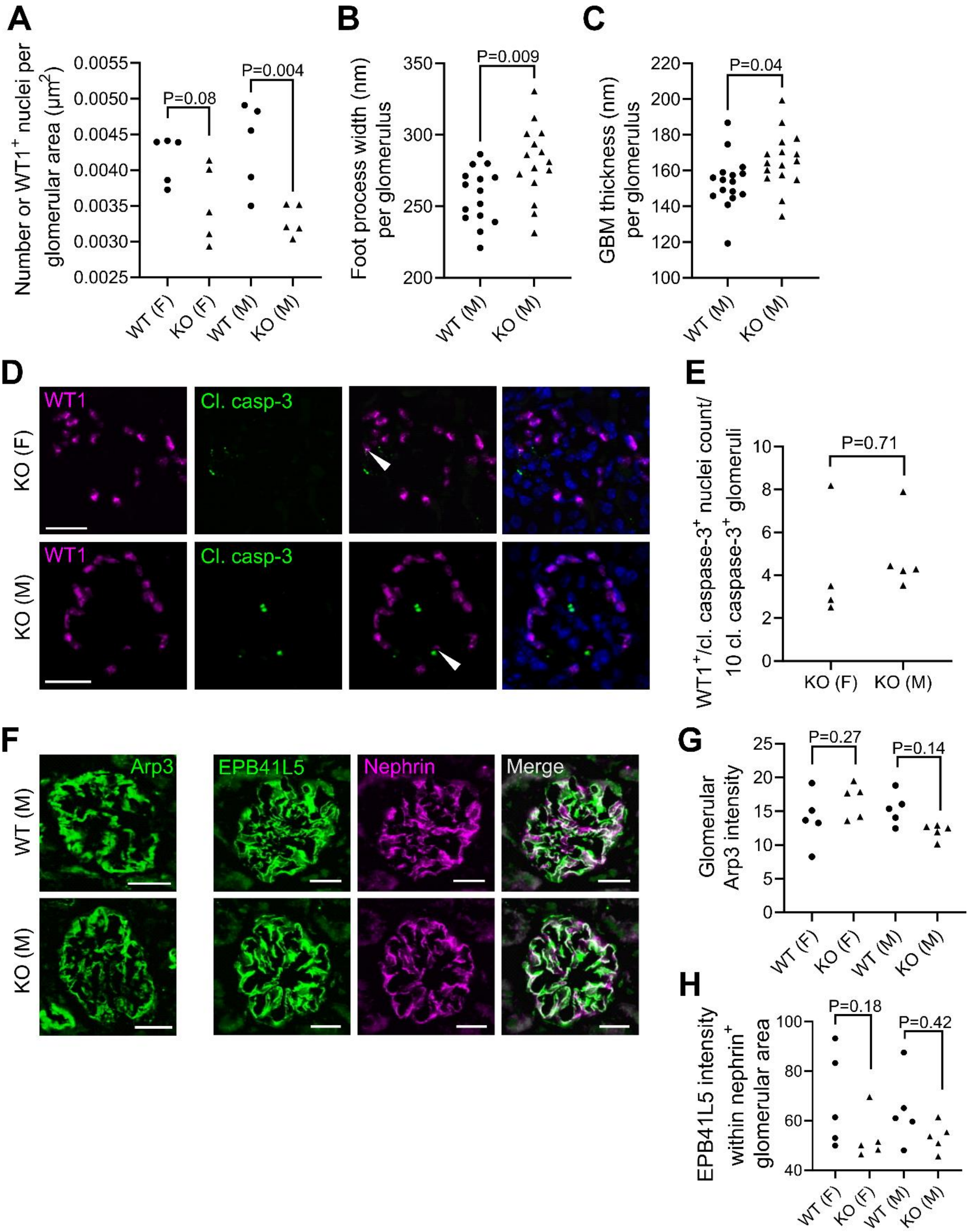

Supplemental Figure 3. Podocyte loss and ultrastructural alterations of the glomeruli were evident in 6-month-old male AdipoR1-KO mice, despite of no change in glomerular Arp3 and EPB41L5 expression levels. (A) The number of podocytes (WT1-positive nuclei), normalized to the glomerular cross-sectional area, was decreased in male AdipoR1-KO mice (n=5 mice/group). (B,C) Mean podocyte foot process width (B) and thickness of the glomerular basement membrane (GBM) (C) were decreased in male AdipoR1-KO mice. Each scatter dot represents one glomerulus (n=3 mice/group, 5 glomeruli/mouse). (D) Representative images of immunofluorescence staining for cleaved caspase-3 and WT1 in the glomeruli of female and

male AdipoR1-KO mice. Scale bar: 25 µm. (E) Quantification of the number of cleaved caspase-3/WT1-double-positive nuclei, represented per 10 cleaved caspase-3-positive glomeruli, showed no difference in the level of podocyte apoptosis between female (n=4) and male (n=5) AdipoR1-KO mice. (F) Representative images of immunofluorescence staining for Arp3, EPB41L5, and nephrin in the glomeruli of male AdipoR1-KO and WT mice. Scale bar: 25 µm. (G,H) Quantification of mean fluorescence intensity for glomerular Arp3 (G), and mean fluorescence intensity for EPB41L5 within the nephrin-positive glomerular area (H), from images as in (F), showed no difference between AdipoR1-KO and WT mice (n=5 mice/group). Scale bar: 25 µm. (A,G,H) Data were assessed by one-way ANOVA with Bonferroni correction. (B,C,E) Data were assessed by Student’s t-test.

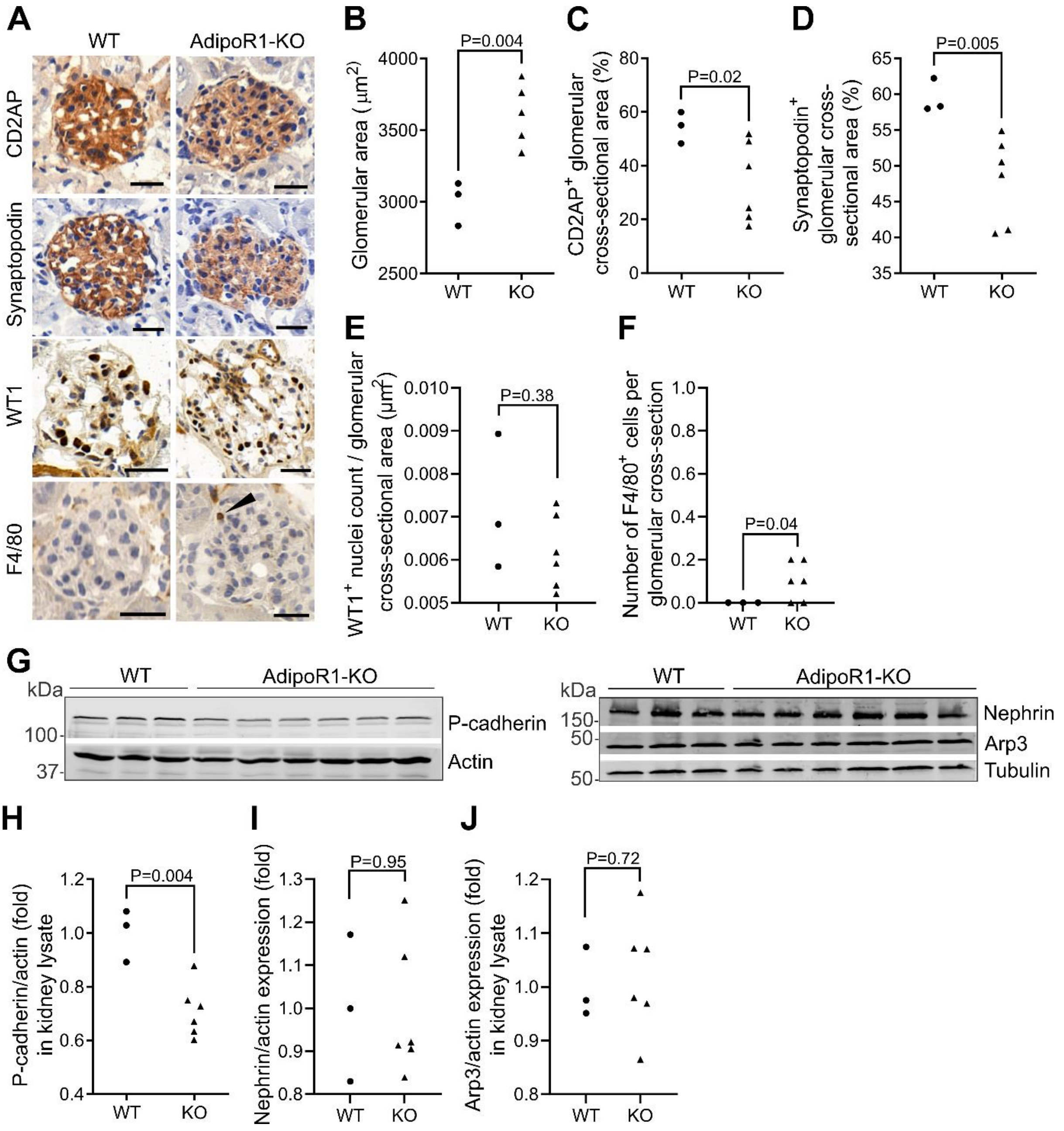

Supplemental Figure 4. AdipoR1-KO mice showed increased glomerular area and decreased expression of podocyte proteins at the age of 2.5 months. (A) Representative images of immunohistochemical staining for CD2AP, synaptopodin, WT1, and F4/80 in kidney sections of 2.5-month-old wildtype (WT) (n=3) and AdipoR1 knockout (AdipoR1-KO) mice (n=6). Scale bar: 25 µm. (B) Glomerular cross-sectional area is increased in AdipoR1-KO mice in comparison to WT mice. (C,D) Quantification of the staining-positive area revealed downregulation of glomerular expression of CD2AP (C) and synaptopodin (D) in 2.5-month-old male AdipoR1-KO mice compared to the WT mice. (E) Quantification of WT1-positive nuclei, normalized to the glomerular cross-sectional area, showed no difference in the number of podocytes in 2.5-month-old male AdipoR1-KO mice compared to WT mice. (F) Quantification of the number of F4/80-positive cells per glomerular cross-section revealed a slight increase in glomerular infiltration of macrophages in 2.5-month-old male AdipoR1-KO mice compared to WT mice. (G) Representative immunoblots for P-cadherin, nephrin and Arp3 in kidney cortical lysates of 2.5-month-old male WT (n=3) and AdipoR1-KO (n=6) mice. (H–J) Quantification of the immunoblot in (G) and normalization to actin or tubulin showed

decreased expression of P-cadherin (H), while no difference in expression of nephrin (I) or Arp3 (J), was observed in the kidney cortex of 2.5-month-old male AdipoR1-KO mice in comparison to WT mice. (B–F, H–J) Data were assessed by Student's t-test.

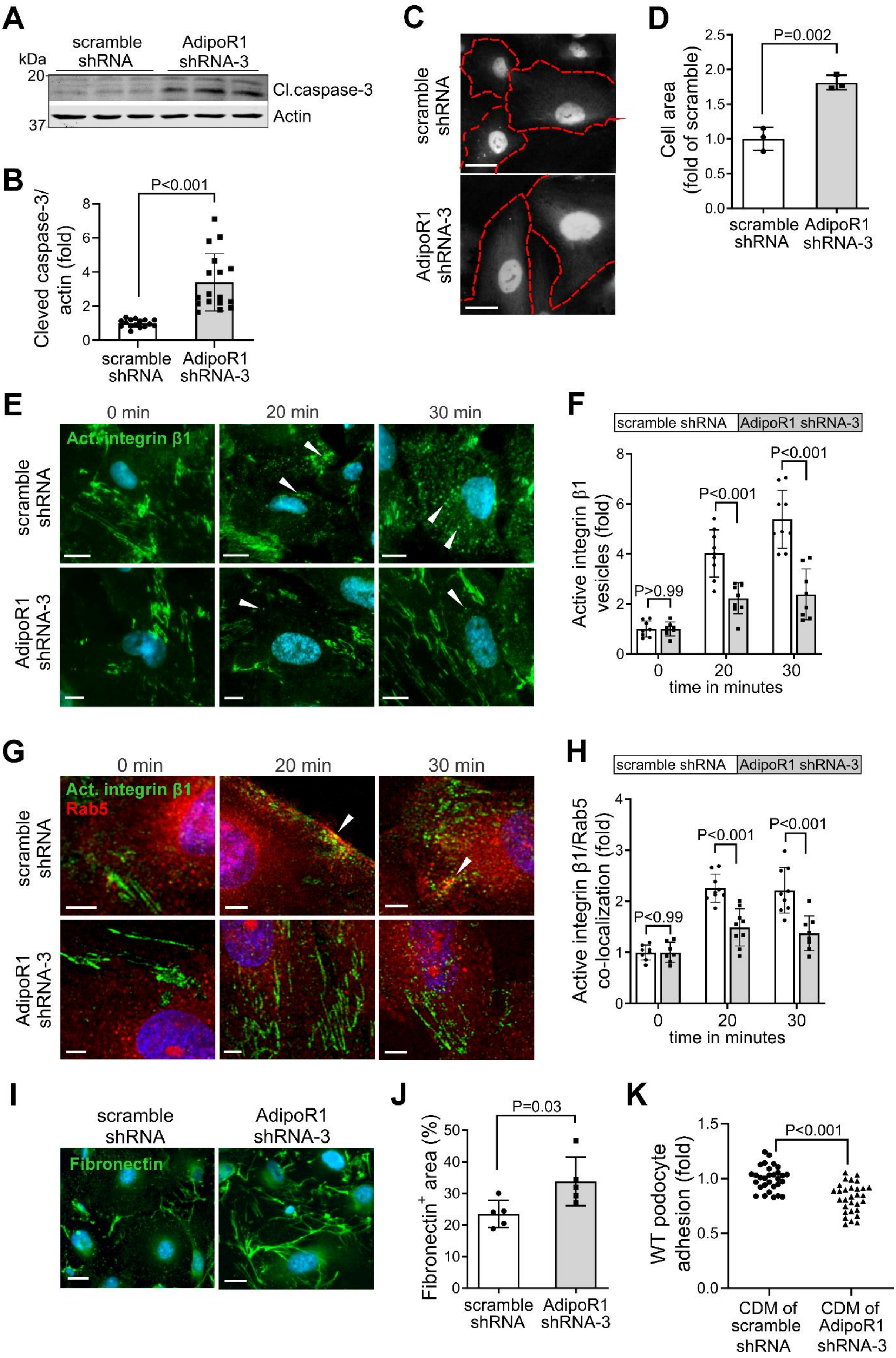

Supplemental Figure 5. Increased apoptosis and cell area, impaired active integrin β1 trafficking, accumulation of fibronectin, and reduced adhesion to cell-derived matrices

in AdipoR1-KD podocytes created using shRNA-3. (A) Representative immunoblots for cleaved caspase-3 in cellular lysates of transient AdipoR1 knockdown (AdipoR1-KD) and control (scramble shRNA) podocytes. (B) The expression level of cleaved caspase-3 normalized to actin was upregulated in transient AdipoR1-KD podocytes compared to control (scramble shRNA) podocytes quantified from immunoblots, as in (A). (C) Representative fluorescence images for stable AdipoR1-KD and scramble (control) podocytes. Cell border is indicated in red. Scale bar: 50 µm. (D) Mean cell area (fold of control) was increased in stable AdipoR1-KD podocytes. (E) Representative immunofluorescence images of surface-labelled active integrin β1 at the plasma membrane (0 min timepoint) and at 20 min and 30 min timepoints following endocytosis (white arrowheads indicate vesicular structures) in stable AdipoR1-KD and control (scramble shRNA) podocytes. Hoechst-stained nuclei are indicated in blue. Scale bar: 20 µm. (F) Quantification of active integrin β1-positive vesicular structures (fold change *vs.* 0 min) revealed a decrease in the endocytosis of active integrin β1 at 20 min and 30 min timepoints in stable AdipoR1-KD podocytes (grey bars) compared to control (scramble shRNA) cells (white bars). (G) Representative confocal images of an immunofluorescence staining for Rab5 (red) and surface-labelled active integrin β1 (green) at 0 min timepoint and after 20 min and 30 min of endocytosis in stable AdipoR1-KD podocytes. Co-localization of Rab5 and active integrin β1 is indicated with white arrowheads. Scale bar: 10 µm. (H) Incidence of co-localization of surface-labelled active integrin β1 and Rab5 (fold change *vs.* 0 min) at 20 min and 30 min timepoints was decreased in stable AdipoR1-KD podocytes (grey bars) compared to control (scramble shRNA) cells (white bars). (I) Representative immunofluorescence staining for fibronectin (green) in stable AdipoR1 knockdown and control (scramble shRNA) podocytes. Nuclei were counterstained with Hoechst stain (blue). Scale bar: 50 µm. (J) Quantification of the fibronectin-positive area from images as in (I) showed an upregulation of fibronectin expression in stable AdipoR1-KD podocytes (grey bars) compared to control (scramble shRNA) podocytes (white bars). (K) Re-adhesion of WT podocytes onto decellularized cell-derived matrices (CDMs) derived from stable AdipoR1-KD podocytes was decreased compared to re-adhesion onto CDMs derived from control (scramble shRNA) podocytes. (B,D,J,K) Data were assessed by Student's t-test. (F,H) Data were assessed by two-way ANOVA with Bonferroni correction.

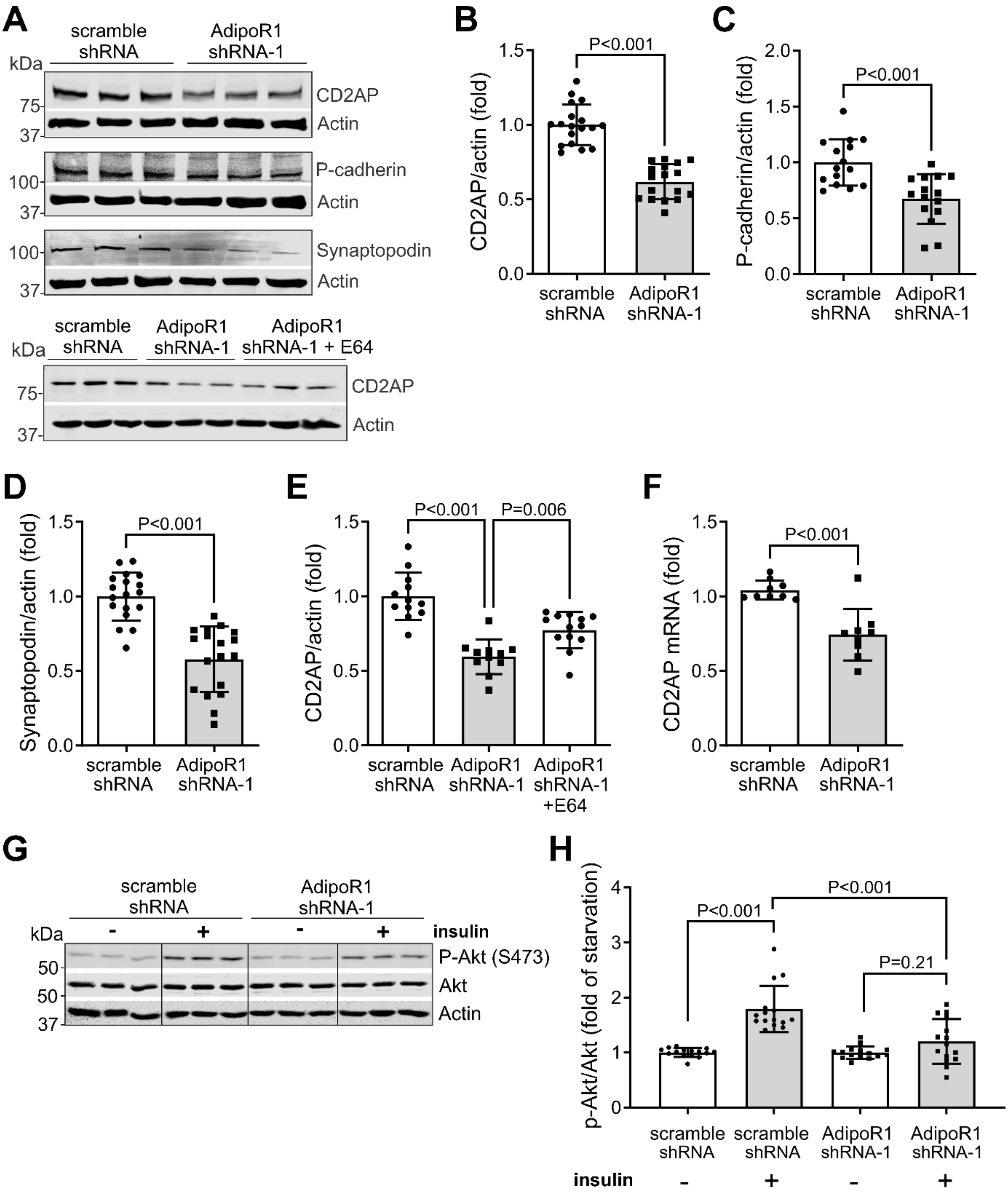

Supplemental Figure 6. Expression of podocyte proteins and insulin-stimulated Akt response were decreased in AdipoR1 knockdown podocytes. (A) Representative immunoblots for CD2AP, P-cadherin, and synaptopodin in cellular lysates of human podocytes transiently transduced with scramble shRNA or AdipoR1 shRNA-1 (AdipoR1-KD). (B–D) The expression levels of podocyte proteins, quantified from immunoblots as in (A), revealed downregulation of CD2AP (B), P-cadherin (C), and synaptopodin (D) in AdipoR1-KD podocytes compared to control (scramble shRNA) podocytes. (E) Quantification of immunoblots as in (A) revealed that treatment with E64 (cathepsin L inhibitor) partially rescued the lowered expression level of full-length CD2AP in AdipoR1-KD podocytes. (F) The expression level of CD2AP mRNA normalized to GAPDH was decreased in transient

AdipoR1-KD podocytes (by shRNA-1) in comparison to control podocytes. (G) Representative immunoblots for phospho-Akt (Ser473) (p-Akt) and total Akt (Akt) in transient AdipoR1-KD (by shRNA-1) and control (scramble shRNA) podocytes with and without insulin stimulation. (H) Insulin stimulation induced Akt phosphorylation in scramble shRNA transduced podocytes, whereas insulin failed to stimulate Akt phosphorylation in AdipoR1-KD podocytes. The graphs show the ratio of phospho-Akt normalized to total Akt, quantified from immunoblots as in (G). (B–H) Data were assessed by two-tailed Student's t-test (2 groups) or one-way ANOVA with Bonferroni correction (3–4 groups).

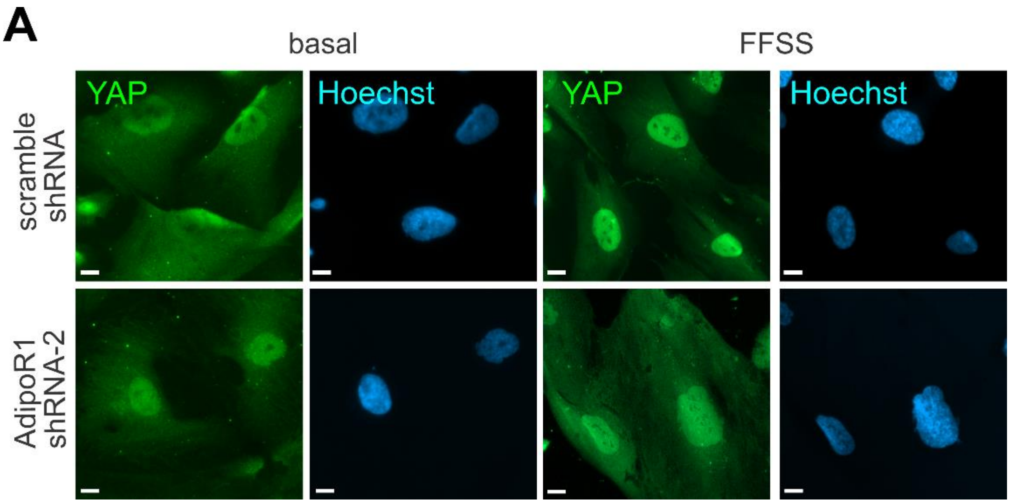

Supplemental Figure 7. AdipoR1 knockdown podocytes show impaired nuclear translocation of YAP in response to fluid flow shear stress. (A) Representative immunofluorescence staining for YAP (green) under basal and fluid flow shear stress (FFSS) conditions in stable AdipoR1-KD (by shRNA-2) and control (scramble shRNA) podocytes. Scale bar: 20 µm.

Supplemental Table 1. Basic characteristics of the study participants who underwent radical nephrectomy. T2D, type 2 diabetes; DKD, diabetic kidney disease (in T2D); BMI, body mass index

**AdipoR1 staining**					
	**Control**		**T2D**		**DKD**
	**Females**	**Males**	**Females**	**Males**	**Females + males**
**n**	17	35	18	28	6
**Age (years)**	71.1 ± 9.9	63.9 ± 11.2	71.5 ± 12.8	66.8 ± 8.7	65.2 ± 6.2
**BMI**	27.7 ± 6.2	26.1 ± 3.9	30.7 ± 6.4	29.3 ± 5.2 [a]	31.6 ± 7.1
[a] P=0.005 versus control males (one-way ANOVA)					

**APPL1 staining**			
	**Control**	**Type 2 diabetes**	
**n (females + males)**	32 (9 + 23)	36 (14+22)	
**Age (years)**	66.59 ± 11.6	68.7 ± 11.8	
**BMI**	27.0 ± 4.6	30.2 ± 5.9 [b]	
[b] P=0.01 versus control (Student’s t-test)			

Supplemental Table 2. Basic characteristics of the study participants whose sera were used in the treatments of immortalized human podocytes. BMI, body mass index.

	**Lean females**	**Lean males**	**Obese females**	**Obese males**
**n**	9	4	5	4
**Age (years)**	45.1 ± 8.3	56.0 ± 3.4	47.6 ± 6.6	42.5 ± 12.6
**BMI**	22.1 ± 2.3	23.3 ± 2.1	44.5 ± 5.1 [c]	37.0 ± 4.8 [d]

[c] P<0.001 versus lean subjects of the same sex (Student's t-test)

[d] P=0.003 versus lean subjects of the same sex (Student's t-test)

Supplemental Table 3. Primary and secondary antibodies used in the study.
IB, immunoblotting; IF, immunofluorescence; IHC, immunohistochemistry; IHC-P, IHC on paraffin sections; NA, not applicable; OCT, Optimal Cutting Temperature compound

Primary antibodies for IB	Manufacturer	Catalog #	RRID	Host	Dilution
Actin	MilliporeSigma, Burlington, MA, USA	A3853	262137	mouse	1:3000
Actin	Abcam, Cambridge, UK	8227	2305186	rabbit	1:3000
Active β-catenin	MilliporeSigma	05-665	309887	mouse	1:500
Active β1 integrin (clone 12G10) [2]	Abcam	30394	775726	mouse	1:750
Arp3	Santa Cruz Biotechnology, Santa Cruz, CA, USA	48344	626700	mouse	1:500
AdipoR1	Immuno-Biological Laboratories (IBL), Takasaki, Japan	18993	N/A	rabbit	1:750
AdipoR1	GeneTex, Irvine, CA, USA	104770	1240425	rabbit	1:500
Akt	R&D systems, Minneapolis, MN, USA	MAB2055	2224581	mouse	1:1000
Phospho-Akt (S473)	Cell Signaling Technology, Beverly, MA, USA	9271	329825	rabbit	1:1000
Arp3	Santa Cruz Biotechnology, Santa Cruz, CA, USA	48344	626700	mouse	1:500
Bax	Santa Cruz Biotechnology	526	2064668	rabbit	1:100
Bcl-2	Santa Cruz Biotechnology	509	626733	mouse	1:100
Cleaved caspase-3	Cell Signaling Technology	9661	2341188	rabbit	1:1000
CD2AP	Santa Cruz Biotechnology	9137	2244374	rabbit	1:750
Paxillin	BD Biosciences, San Diego, CA, USA	610051	397464	mouse	1:2000
P-Paxillin (Y31)	Abcam	ab4832	304668	rabbit	1:1000

P-Cadherin	Novus Biologicals, Littleton, CO, USA	59222	11045547	rabbit	1:500
P-Cadherin	BD Biosciences	610228	2077667	mouse	1:500
Synaptopodin	Santa Cruz Biotechnology	515842	N/A	mouse	1:200
Tubulin	MilliporeSigma	T6199	477583	mouse	1:3000

**Primary antibodies for IHC/IF**	**Manufacturer**	**Catalog # / RRID**	**Host**	**Dilution**	**Retrieval buffer**	**Detection kit**
Adipophilin[3]	Invitrogen	PA1-16972 / 2223607	rabbit	1:500	Tris-EDTA	Brightvision
AdipoR1	GeneTex, Irvine, CA, USA	104770 / 1240425	rabbit	1:2000	Envision	EnVision FLEX+
APPL1	Genetex	115786 / 10619598	rabbit	1:200	Citrate	Brightvision
APPL1	Cell Signaling Technology	3858 / 2056989	rabbit	1:800	Envision	Brightvision
CD2AP	Santa Cruz Biotechnology	9137 / 2244374	rabbit	1:150 (OCT), 1:800 (IHC-P)	Envision	EnVision+ / Brightvision
Cleaved caspase-3	Cell Signaling Technology	9661 / 2341188	rabbit	1:100	Envision / Tris-EDTA	Brightvision
F4/80	Cell Signaling Technology	70076 / 2799771	rabbit	1/300 (OCT), 1/500 (IHC-P)	Envision	Brightvision
Fibronectin	Abcam	ab299 / 303474	rabbit	1:3000	Envision	Brightvision
Synaptopodin	Progen, Heidelberg, Germany	61094 / 2335879	mouse	1:50 (OCT), 1/600 (IHC-P)	Envision	EnVision+ / Brightvision
TGFβ1	Abcam	ab215715 / 2893156	rabbit	1:300	Envision	Brightvision
WT1	Santa Cruz Biotechnology	SC-192 / NA	rabbit	1:300 (OCT), 1:1000 (IHC-P)	Envision	Brightvision
WT1	BiCell, Maryland Heights, MO, USA	00180 / NA	rat	1:500	Tris-EDTA	Brightvision

Primary antibodies for IF	Manufacturer	Catalog #	RRID	Host	Dilution
Active β1 integrin (clone 12G10; against human) [2]	Abcam	30394	775726	mouse	1:100
Active β1 integrin (clone 9EG7; against mouse)	BD Biosciences	550531	393729	rat	1:50
APPL1	Cell Signaling Technology	3858	2056989	rabbit	1:100
Arp3	Santa Cruz Biotechnology, Santa Cruz, CA, USA	48344	626700	mouse	1:300
EPB41L5	BiCell Scientific, Maryland Heights, MO, USA	31245	N/A	rat	1:100
Fibronectin	Abcam	ab299	303474	rabbit	1:500
Nephrin	Progen	GP-N2	2904121	guinea pig	1:700
Paxillin	BD Biosciences, San Diego, CA, USA	610051	397464	mouse	1:500
Rab5	Cell Signaling Technology	3547	2300649	rabbit	1:75
YAP	Cell Signaling Technology	14074	2650491	rabbit	1:100
WT1	Millipore, Burlington, MA, USA	05-753	309966	mouse	1:300

Secondary antibodies	Manufacturer	Catalog #	RRID	Host	Dilution
IRDye 680 anti-rabbit IgG	LI-COR, Lincoln, NE, USA	926-32221	621841	goat	1:10,000
IRDye 680RD anti-mouse IgG	LI-COR	926-68072	10953628	donkey	1:10,000
IRDye 800CW anti-rabbit IgG	LI-COR	926-32213	621848	donkey	1:10,000
IRDye 800CW anti-mouse IgG	LI-COR	926-32212	621847	donkey	1:10,000
AlexaFluor 594 anti-rabbit IgG	Invitrogen	A-21207	141637	donkey	1:1000

AlexaFluor 488 anti-mouse IgG	Invitrogen	A-21202	141607	donkey	1:1000
TSA-Alexa Fluor 488	Invitrogen	B40953	NA	NA	1:100
CF594 anti-guinea pig IgG	Sigma-Aldrich	SAB4600096	2728625	donkey	1:1000
AlexaFluor 488 anti-rat IgG	Invitrogen	A21208	2535794	donkey	1:1000
AlexaFluor 488 anti-rabbit IgG	Invitrogen	A21206	2535792	donkey	1:1000
AlexaFluor 488 anti-mouse IgG	Invitrogen	A-21121	141514	goat	1:1000
AlexaFluor 555 anti-rat IgG	Invitrogen	A21434	2535855	goat	1:1000
AlexaFluor 488 anti-guinea pig IgG	Invitrogen	A11073	2534117	goat	1:1000
Alexa Fluor Plus 647 anti-rat IgG	Invitrogen	A48265	2895299	goat	1:300

Supplemental Methods

Double immunofluorescence staining on paraffin sections
Cleaved caspase-3/WT1 double-staining was carried out on kidney paraffin sections of AdipoR1-KO mice. Antigen retrieval of deparaffinized kidney sections was performed in 10mM Tris-1mM EDTA, pH 9 (20 min 99°C). Sections were blocked with 10% goat serum in TBS+Tween and incubated with indicated primary antibodies for 1 h at room temperature. Tyramide signal amplification (TSA488) was used for the detection of cleaved caspase-3. Antibody details are described in Supplemental Table 3. To quantify the number of apoptotic podocytes, at least 20 glomeruli that were positive for cleaved caspase-3 were annotated, and the number of cleaved caspase-3/WT1-double-positive nuclei was presented per 10 glomeruli.

2.5-month-old male AdipoR1-KO mice
The mouse strain, B6.129P2-Adipor1tm1Dgen/Mmnc, RRID: MMRRC_011599-UNC, was obtained from the Mutant Mouse Resource and Research Center (MMRRC) at the University of North Carolina at Chapel Hill, an NIH-funded strain repository, and purchased from The Jackson Laboratories (Bar Harbor, ME). Homozygous AdipoR1-KO (AdipoR1–/–) mice were created by interbreeding of the heterozygous mice. Littermate wildtype (WT) mice were used as controls. The animal study was conducted according to the Guide for the Care and Use of Laboratory Animals published by the US National Institutes of Health.

The kidneys were harvested and snap-frozen at the age of 8–11 weeks (WT littermates: 9.3±0.6 weeks; AdipoR1-KO: 9.1±1.3 weeks). Frozen kidney tissue was embedded in Tissue-Tek OCT media (Sakura Finetek, Torrance, CA, USA) and snap-frozen using dry ice. Alternatively, kidney cortex was homogenized with a Dounce homogenizer in RIPA buffer supplemented with 1x EDTA-free cOmplete protease inhibitor cocktail (Roche, Mannheim, Germany), 50 mM NaF and 1 mM Na_3VO_4.

Insulin stimulation of immortalized podocytes
For insulin stimulation, podocytes were starved in the RPMI1640 medium, without FBS and Insulin-Transferrin-Selenium supplement, for 16 h, followed by stimulation with 200 nM insulin (Actrapid, Novo Nordisk, Copenhagen, Denmark) for 15 min.

Gene expression analysis
Total cellular RNA was extracted with a RNeasy mini kit (Qiagen, Hilden, Germany), and cDNA was synthesized with a High-Capacity RNA-to-cDNA kit (Applied Biosystems, Foster City, CA, USA). Quantitative RT-PCR was performed with the SensiFAST SYBR Lo-ROX kit (Meridian Bioscience, Cincinnati, OH, USA). The primer pair used for the detection of CD2AP was 5’AGGCTGGTGGAGTGGAAC 3' (forward) and 5’CAGAGAAGGTATAGGTGAAGTAGG 3' (reverse). GAPDH was used as a reference gene for normalization. qPCR was carried out in a C1000 Touch Thermal Cycler CFX96 or CFX384 Real-Time System (Bio-Rad Laboratories, Hercules, CA, USA). The expression level was quantified with Bio-Rad CFX Maestro software (version 4.1), using the ΔΔCq method.

References

1. Pan Y, Jiang S, Hou Q, et al. Dissection of Glomerular Transcriptional Profile in Patients With Diabetic Nephropathy: SRGAP2a Protects Podocyte Structure and Function. Diabetes. 2017;67(4):717-730

2. Mould AP, Garratt AN, Askari JA, et al. Identification of a novel anti-integrin monoclonal antibody that recognises a ligand-induced binding site epitope on the β1 subunit. FEBS Lett. 1995;363(1):118-122

3. Herman-Edelstein M, Scherzer P, Tobar A, et al. Altered renal lipid metabolism and renal lipid accumulation in human diabetic nephropathy. J Lipid Res. 2014;55(3):561-572
