# Supplementary material for: Integrin Trafficking, Fibronectin Architecture, and Glomerular Injury upon Adiponectin Receptor 1 Depletion
Source: J Am Soc Nephrol. 2025 Jan 28;36(5):825–44. doi: 10.1681/ASN.0000000611 (PMC12059104; doi:10.1681/ASN.0000000611)
Supplement: Supplementary file 4 [file jasn-36-825-s004.pdf]

ASN American Society of Nephrology · JASN Journal of the American Society of Nephrology · KIDNEY360 Accessing Our World From Every Angle · CJASN Clinical Journal of the American Society of Nephrology

ASN Journal Disclosure Form

As per ASN journal policy, I have disclosed any financial relationships or commitments I have held in the past 36 months as included below. I have listed my Current Employer below to indicate there is a relationship requiring disclosure. If no relationship exists, my Current Employer is not listed.

H. Bugger reports the following:
Consultancy: RobotDreams; Honoraria: RobotDreams; Bayer; Vifor; Corvia Medical; Boehringer; Advisory or Leadership Role: Boehringer, RobotDreams; and Speakers Bureau: Corvia Medical.

I understand that the information above will be published within the journal article, if accepted, and that failure to comply and/or to accurately and completely report the potential financial conflicts of interest could lead to the following: 1) Prior to publication, article rejection, or 2) Post-publication, sanctions ranging from, but not limited to, issuing a correction, reporting the inaccurate information to the authors' institution, banning authors from submitting work to ASN journals for varying lengths of time, and/or retraction of the published work.

Name: Heiko Bugger
Manuscript ID: JASN-2023-001160R1
Manuscript Title: AdipoR1 depletion induces glomerular injury and disturbs integrin β1 trafficking and fibronectin architecture in podocytes in vitro
Date of Completion: July 12, 2024
Disclosure Updated Date: July 12, 2024

ASN American Society of Nephrology | JASN Journal of the American Society of Nephrology | KIDNEY360 Accessing Our World From Every Angle | CJASN Clinical Journal of the American Society of Nephrology

ASN Journal Disclosure Form

As per ASN journal policy, I have disclosed any financial relationships or commitments I have held in the past 36 months as included below. I have listed my Current Employer below to indicate there is a relationship requiring disclosure. If no relationship exists, my Current Employer is not listed.

A. Hau reports the following:
Employer: University of Helsinki; and Ownership Interest: Sampo Oyj; Coinbase Global Inc; Mandatum oyj; Nvidia Corp.

I understand that the information above will be published within the journal article, if accepted, and that failure to comply and/or to accurately and completely report the potential financial conflicts of interest could lead to the following: 1) Prior to publication, article rejection, or 2) Post-publication, sanctions ranging from, but not limited to, issuing a correction, reporting the inaccurate information to the authors' institution, banning authors from submitting work to ASN journals for varying lengths of time, and/or retraction of the published work.

Name: Annika Hau
Manuscript ID: JASN-2023-001160R2
Manuscript Title: Integrin trafficking, fibronectin architecture and glomerular injury upon AdipoR1 depletion
Date of Completion: December 16, 2024
Disclosure Updated Date: December 16, 2024

ASN American Society of Nephrology | JASN Journal of the American Society of Nephrology | KIDNEY360 Accessing Our World From Every Angle | CJASN Clinical Journal of the American Society of Nephrology

ASN Journal Disclosure Form

As per ASN journal policy, I have disclosed any financial relationships or commitments I have held in the past 36 months as included below. I have listed my Current Employer below to indicate there is a relationship requiring disclosure. If no relationship exists, my Current Employer is not listed.

M. Karhe reports the following:
Employer: University of Helsinki; Vitabalans Oy;

I understand that the information above will be published within the journal article, if accepted, and that failure to comply and/or to accurately and completely report the potential financial conflicts of interest could lead to the following: 1) Prior to publication, article rejection, or 2) Post-publication, sanctions ranging from, but not limited to, issuing a correction, reporting the inaccurate information to the authors' institution, banning authors from submitting work to ASN journals for varying lengths of time, and/or retraction of the published work.

Name: Minna Karhe
Manuscript ID: JASN-2023-001160R1
Manuscript Title: AdipoR1 depletion induces glomerular injury and disturbs integrin β1 trafficking and fibronectin architecture in podocytes in vitro
Date of Completion: July 12, 2024
Disclosure Updated Date: July 12, 2024

ASN American Society of Nephrology
JASN Journal of the American Society of Nephrology
KIDNEY360 Accessing Our World From Every Angle
CJASN Clinical Journal of the American Society of Nephrology

ASN Journal Disclosure Form

As per ASN journal policy, I have disclosed any financial relationships or commitments I have held in the past 36 months as included below. I have listed my Current Employer below to indicate there is a relationship requiring disclosure. If no relationship exists, my Current Employer is not listed.

E. Lehtonen reports the following:
Employer: University of Helsinki

I understand that the information above will be published within the journal article, if accepted, and that failure to comply and/or to accurately and completely report the potential financial conflicts of interest could lead to the following: 1) Prior to publication, article rejection, or 2) Post-publication, sanctions ranging from, but not limited to, issuing a correction, reporting the inaccurate information to the authors' institution, banning authors from submitting work to ASN journals for varying lengths of time, and/or retraction of the published work.

Name: Eero Lehtonen
Manuscript ID: JASN-2023-001160R2
Manuscript Title: Integrin trafficking, fibronectin architecture and glomerular injury upon AdipoR1 depletion
Date of Completion: December 15, 2024
Disclosure Updated Date: December 15, 2024

ASN
American Society of Nephrology
JASN
Journal of the American Society of Nephrology
KIDNEY360
Accessing Our World From Every Angle
CJASN
Clinical Journal of the American Society of Nephrology

ASN Journal Disclosure Form

As per ASN journal policy, I have disclosed any financial relationships or commitments I have held in the past 36 months as included below. I have listed my Current Employer below to indicate there is a relationship requiring disclosure. If no relationship exists, my Current Employer is not listed.

S. Lehtonen reports the following:
Employer: University of Helsinki; Ownership Interest: Sebastian Ventures Corp.; and Research Funding: Novo Nordisk Foundation (I have received funding for a research project from the Novo Nordisk Foundation, but the funding was not for the project presented in the submitted JASN manuscript).

I understand that the information above will be published within the journal article, if accepted, and that failure to comply and/or to accurately and completely report the potential financial conflicts of interest could lead to the following: 1) Prior to publication, article rejection, or 2) Post-publication, sanctions ranging from, but not limited to, issuing a correction, reporting the inaccurate information to the authors' institution, banning authors from submitting work to ASN journals for varying lengths of time, and/or retraction of the published work.

Name: Sanna H. Lehtonen
Manuscript ID: JASN-2023-001160R1
Manuscript Title: AdipoR1 depletion induces glomerular injury and disturbs integrin β1 trafficking and fibronectin architecture in podocytes in vitro
Date of Completion: July 12, 2024
Disclosure Updated Date: July 12, 2024

ASN American Society of Nephrology · JASN Journal of the American Society of Nephrology · KIDNEY360 Accessing Our World From Every Angle · CJASN Clinical Journal of the American Society of Nephrology

ASN Journal Disclosure Form

As per ASN journal policy, I have disclosed any financial relationships or commitments I have held in the past 36 months as included below. I have listed my Current Employer below to indicate there is a relationship requiring disclosure. If no relationship exists, my Current Employer is not listed.

D. Lewandowski reports the following:
Employer: University of California Irvine

I understand that the information above will be published within the journal article, if accepted, and that failure to comply and/or to accurately and completely report the potential financial conflicts of interest could lead to the following: 1) Prior to publication, article rejection, or 2) Post-publication, sanctions ranging from, but not limited to, issuing a correction, reporting the inaccurate information to the authors' institution, banning authors from submitting work to ASN journals for varying lengths of time, and/or retraction of the published work.

Name: Dominik Lewandowski
Manuscript ID: JASN-2023-001160R2
Manuscript Title: Integrin trafficking, fibronectin architecture and glomerular injury upon AdipoR1 depletion
Date of Completion: December 16, 2024
Disclosure Updated Date: December 16, 2024

ASN American Society of Nephrology JASN Journal of the American Society of Nephrology KIDNEY360 Accessing Our World From Every Angle CJASN Clinical Journal of the American Society of Nephrology

ASN Journal Disclosure Form

As per ASN journal policy, I have disclosed any financial relationships or commitments I have held in the past 36 months as included below. I have listed my Current Employer below to indicate there is a relationship requiring disclosure. If no relationship exists, my Current Employer is not listed.

S. Lindfors reports the following:
Employer: ART Sofware Oy, Finland; and Research Funding: Finnish Diabetes Research Foundation.

I understand that the information above will be published within the journal article, if accepted, and that failure to comply and/or to accurately and completely report the potential financial conflicts of interest could lead to the following: 1) Prior to publication, article rejection, or 2) Post-publication, sanctions ranging from, but not limited to, issuing a correction, reporting the inaccurate information to the authors' institution, banning authors from submitting work to ASN journals for varying lengths of time, and/or retraction of the published work.

Name: Sonja Lindfors
Manuscript ID: JASN-2023-001160R2
Manuscript Title: Integrin trafficking, fibronectin architecture and glomerular injury upon AdipoR1 depletion.
Date of Completion: December 16, 2024
Disclosure Updated Date: December 16, 2024

ASN American Society of Nephrology | JASN Journal of the American Society of Nephrology | KIDNEY360 Accessing Our World From Every Angle | CJASN Clinical Journal of the American Society of Nephrology

ASN Journal Disclosure Form

As per ASN journal policy, I have disclosed any financial relationships or commitments I have held in the past 36 months as included below. I have listed my Current Employer below to indicate there is a relationship requiring disclosure. If no relationship exists, my Current Employer is not listed.

V. Majaniemi has nothing to disclose.

I understand that the information above will be published within the journal article, if accepted, and that failure to comply and/or to accurately and completely report the potential financial conflicts of interest could lead to the following: 1) Prior to publication, article rejection, or 2) Post-publication, sanctions ranging from, but not limited to, issuing a correction, reporting the inaccurate information to the authors' institution, banning authors from submitting work to ASN journals for varying lengths of time, and/or retraction of the published work.

Name: Ville Majaniemi
Manuscript ID: JASN-2023-001160R1
Manuscript Title: AdipoR1 depletion induces glomerular injury and disturbs integrin β1 trafficking and fibronectin architecture in podocytes in vitro
Date of Completion: July 22, 2024
Disclosure Updated Date: July 22, 2024

ASN Journal Disclosure Form

As per ASN journal policy, I have disclosed any financial relationships or commitments I have held in the past 36 months as included below. I have listed my Current Employer below to indicate there is a relationship requiring disclosure. If no relationship exists, my Current Employer is not listed.

T. Mirtti reports the following:
Consultancy: Aiforia Technologies Plc

I understand that the information above will be published within the journal article, if accepted, and that failure to comply and/or to accurately and completely report the potential financial conflicts of interest could lead to the following: 1) Prior to publication, article rejection, or 2) Post-publication, sanctions ranging from, but not limited to, issuing a correction, reporting the inaccurate information to the authors' institution, banning authors from submitting work to ASN journals for varying lengths of time, and/or retraction of the published work.

Name: Tuomas Mirtti
Manuscript ID: JASN-2023-001160R3
Manuscript Title: Integrin Trafficking, Fibronectin Architecture, and Glomerular Injury upon AdipoR1 Depletion
Date of Completion: January 4, 2025
Disclosure Updated Date: January 4, 2025

ASN American Society of Nephrology | JASN Journal of the American Society of Nephrology | KIDNEY360 Accessing Our World From Every Angle | CJASN Clinical Journal of the American Society of Nephrology

ASN Journal Disclosure Form

As per ASN journal policy, I have disclosed any financial relationships or commitments I have held in the past 36 months as included below. I have listed my Current Employer below to indicate there is a relationship requiring disclosure. If no relationship exists, my Current Employer is not listed.

J. Naams has nothing to disclose.

I understand that the information above will be published within the journal article, if accepted, and that failure to comply and/or to accurately and completely report the potential financial conflicts of interest could lead to the following: 1) Prior to publication, article rejection, or 2) Post-publication, sanctions ranging from, but not limited to, issuing a correction, reporting the inaccurate information to the authors' institution, banning authors from submitting work to ASN journals for varying lengths of time, and/or retraction of the published work.

Name: Jette-Britt Naams
Manuscript ID: JASN-2023-001160R1
Manuscript Title: AdipoR1 depletion induces glomerular injury and disturbs integrin β1 trafficking and fibronectin architecture in podocytes in vitro
Date of Completion: July 22, 2024
Disclosure Updated Date: July 22, 2024

ASN American Society of Nephrology · JASN Journal of the American Society of Nephrology · KIDNEY360 Accessing Our World From Every Angle · CJASN Clinical Journal of the American Society of Nephrology

ASN Journal Disclosure Form

As per ASN journal policy, I have disclosed any financial relationships or commitments I have held in the past 36 months as included below. I have listed my Current Employer below to indicate there is a relationship requiring disclosure. If no relationship exists, my Current Employer is not listed.

H. Nisen has nothing to disclose.

I understand that the information above will be published within the journal article, if accepted, and that failure to comply and/or to accurately and completely report the potential financial conflicts of interest could lead to the following: 1) Prior to publication, article rejection, or 2) Post-publication, sanctions ranging from, but not limited to, issuing a correction, reporting the inaccurate information to the authors' institution, banning authors from submitting work to ASN journals for varying lengths of time, and/or retraction of the published work.

Name: Harry Nisen
Manuscript ID: JASN-2023-001160R1
Manuscript Title: AdipoR1 depletion induces glomerular injury and disturbs integrin β1 trafficking and fibronectin architecture in podocytes in vitro
Date of Completion: July 17, 2024
Disclosure Updated Date: July 17, 2024

ASN American Society of Nephrology JASN Journal of the American Society of Nephrology KIDNEY360 Accessing Our World From Every Angle CJASN Clinical Journal of the American Society of Nephrology

ASN Journal Disclosure Form

As per ASN journal policy, I have disclosed any financial relationships or commitments I have held in the past 36 months as included below. I have listed my Current Employer below to indicate there is a relationship requiring disclosure. If no relationship exists, my Current Employer is not listed.

K. Palczewski reports the following:
Employer: University of California, Irvine,Department of Physiology and Biophysics

I understand that the information above will be published within the journal article, if accepted, and that failure to comply and/or to accurately and completely report the potential financial conflicts of interest could lead to the following: 1) Prior to publication, article rejection, or 2) Post-publication, sanctions ranging from, but not limited to, issuing a correction, reporting the inaccurate information to the authors' institution, banning authors from submitting work to ASN journals for varying lengths of time, and/or retraction of the published work.

Name: Krzysztof Palczewski
Manuscript ID: JASN-2023-001160R2
Manuscript Title: Integrin trafficking, fibronectin architecture and glomerular injury upon AdipoR1 depletion
Date of Completion: January 3, 2025
Disclosure Updated Date: January 3, 2025

ASN American Society of Nephrology
JASN Journal of the American Society of Nephrology
KIDNEY360 Accessing Our World From Every Angle
CJASN Clinical Journal of the American Society of Nephrology

ASN Journal Disclosure Form

As per ASN journal policy, I have disclosed any financial relationships or commitments I have held in the past 36 months as included below. I have listed my Current Employer below to indicate there is a relationship requiring disclosure. If no relationship exists, my Current Employer is not listed.

K. Pfeil reports the following:
Employer: Medical University of Graz

I understand that the information above will be published within the journal article, if accepted, and that failure to comply and/or to accurately and completely report the potential financial conflicts of interest could lead to the following: 1) Prior to publication, article rejection, or 2) Post-publication, sanctions ranging from, but not limited to, issuing a correction, reporting the inaccurate information to the authors' institution, banning authors from submitting work to ASN journals for varying lengths of time, and/or retraction of the published work.

Name: Katharina Pfeil
Manuscript ID: JASN-2023-001160R2
Manuscript Title: Integrin Trafficking, Fibronectin Architecture, and Glomerular Injury upon AdipoR1 Depletion
Date of Completion: January 2, 2025
Disclosure Updated Date: January 2, 2025

ASN American Society of Nephrology | JASN Journal of the American Society of Nephrology | KIDNEY360 Accessing Our World From Every Angle | CJASN Clinical Journal of the American Society of Nephrology

ASN Journal Disclosure Form

As per ASN journal policy, I have disclosed any financial relationships or commitments I have held in the past 36 months as included below. I have listed my Current Employer below to indicate there is a relationship requiring disclosure. If no relationship exists, my Current Employer is not listed.

K. Pietiläinen reports the following:
Employer: University of Helsinki; Research Funding: Novo Nordisk; Honoraria: Novo Nordisk, Orion, AstraZeneca, Eli Lilly; Advisory or Leadership Role: Novo Nordisk, Eli Lilly; and Speakers Bureau: Novo Nordisk, Eli Lilly, AstraZeneca.

I understand that the information above will be published within the journal article, if accepted, and that failure to comply and/or to accurately and completely report the potential financial conflicts of interest could lead to the following: 1) Prior to publication, article rejection, or 2) Post-publication, sanctions ranging from, but not limited to, issuing a correction, reporting the inaccurate information to the authors' institution, banning authors from submitting work to ASN journals for varying lengths of time, and/or retraction of the published work.

Name: Kirsi H. Pietiläinen
Manuscript ID: JASN-2023-001160R2
Manuscript Title: Integrin Trafficking, Fibronectin Architecture, and Glomerular Injury upon AdipoR1 Depletion
Date of Completion: December 27, 2024
Disclosure Updated Date: December 27, 2024

ASN American Society of Nephrology
JASN Journal of the American Society of Nephrology
KIDNEY360 Accessing Our World From Every Angle
CJASN Clinical Journal of the American Society of Nephrology

ASN Journal Disclosure Form

As per ASN journal policy, I have disclosed any financial relationships or commitments I have held in the past 36 months as included below. I have listed my Current Employer below to indicate there is a relationship requiring disclosure. If no relationship exists, my Current Employer is not listed.

L. Saikko has nothing to disclose.

I understand that the information above will be published within the journal article, if accepted, and that failure to comply and/or to accurately and completely report the potential financial conflicts of interest could lead to the following: 1) Prior to publication, article rejection, or 2) Post-publication, sanctions ranging from, but not limited to, issuing a correction, reporting the inaccurate information to the authors' institution, banning authors from submitting work to ASN journals for varying lengths of time, and/or retraction of the published work.

Name: Leena Ls Saikko
Manuscript ID: JASN-2023-001160R1
Manuscript Title: AdipoR1 depletion induces glomerular injury and disturbs integrin beta1 trakkicking and fibronectin architecture in podocytes in vitro
Date of Completion: July 20, 2024
Disclosure Updated Date: July 20, 2024

ASN American Society of Nephrology | JASN Journal of the American Society of Nephrology | KIDNEY360 Accessing Our World From Every Angle | CJASN Clinical Journal of the American Society of Nephrology

ASN Journal Disclosure Form

As per ASN journal policy, I have disclosed any financial relationships or commitments I have held in the past 36 months as included below. I have listed my Current Employer below to indicate there is a relationship requiring disclosure. If no relationship exists, my Current Employer is not listed.

M. Saleem reports the following:
Employer: University of Bristol; Consultancy: Travere Therapeutics; Mission Therapeutics; Pfizer; Confo Therapeutics; Novartis; Santhera; Ownership Interest: Stock options in Purespring Therapeutics; Research Funding: UCB; Evotec; Travere; Honoraria: Purespring Therapeutics - Director and Chief Scientific Officer; Travere Therapeutics;; Patents or Royalties: Purespring Therapeutics; University of Bristol; and Advisory or Leadership Role: Director and Board Member, Purespring Therapeutics; Director, Kidney Research UK.

I understand that the information above will be published within the journal article, if accepted, and that failure to comply and/or to accurately and completely report the potential financial conflicts of interest could lead to the following: 1) Prior to publication, article rejection, or 2) Post-publication, sanctions ranging from, but not limited to, issuing a correction, reporting the inaccurate information to the authors' institution, banning authors from submitting work to ASN journals for varying lengths of time, and/or retraction of the published work.

Name: Moin A. Saleem
Manuscript ID: JASN-2023-001160R2
Manuscript Title: Integrin trafficking, fibronectin architecture and glomerular injury upon AdipoR1 depletion
Date of Completion: December 15, 2024
Disclosure Updated Date: November 25, 2024

ASN
American Society of Nephrology
JASN
Journal of the American Society of Nephrology
KIDNEY360
Accessing Our World From Every Angle
CJASN
Clinical Journal of the American Society of Nephrology

ASN Journal Disclosure Form

As per ASN journal policy, I have disclosed any financial relationships or commitments I have held in the past 36 months as included below. I have listed my Current Employer below to indicate there is a relationship requiring disclosure. If no relationship exists, my Current Employer is not listed.

C. Schmotz reports the following:
Employer: University of Helsinki; and Research Funding: Novo Nordisk Foundation (my PI, Prof. S. Lehtonen, received funding from this foundation for a research project I am involved in, but this funding does not concern the project presented in the submitted JASN manuscript).

I understand that the information above will be published within the journal article, if accepted, and that failure to comply and/or to accurately and completely report the potential financial conflicts of interest could lead to the following: 1) Prior to publication, article rejection, or 2) Post-publication, sanctions ranging from, but not limited to, issuing a correction, reporting the inaccurate information to the authors' institution, banning authors from submitting work to ASN journals for varying lengths of time, and/or retraction of the published work.

Name: Constanze Schmotz
Manuscript ID: JASN-2023-001160R2
Manuscript Title: "Integrin trafficking, fibronectin architecture and glomerular injury upon AdipoR1 depletion,"
Date of Completion: December 15, 2024
Disclosure Updated Date: July 13, 2024

ASN American Society of Nephrology
JASN Journal of the American Society of Nephrology
KIDNEY360 Accessing Our World From Every Angle
CJASN Clinical Journal of the American Society of Nephrology

ASN Journal Disclosure Form

As per ASN journal policy, I have disclosed any financial relationships or commitments I have held in the past 36 months as included below. I have listed my Current Employer below to indicate there is a relationship requiring disclosure. If no relationship exists, my Current Employer is not listed.

J. Tienari reports the following:
Employer: Helsinki University Hospital district

I understand that the information above will be published within the journal article, if accepted, and that failure to comply and/or to accurately and completely report the potential financial conflicts of interest could lead to the following: 1) Prior to publication, article rejection, or 2) Post-publication, sanctions ranging from, but not limited to, issuing a correction, reporting the inaccurate information to the authors' institution, banning authors from submitting work to ASN journals for varying lengths of time, and/or retraction of the published work.

Name: Jukka Pekka Tienari
Manuscript ID: ASN-2023-001160R2
Manuscript Title: Integrin trafficking, fibronectin architecture and glomerular injury upon AdipoR1 depletion.
Date of Completion: December 18, 2024
Disclosure Updated Date: July 12, 2024
